# Hypoxia-induced miR-210 modulates the inflammatory response and fibrosis upon acute ischemia

**DOI:** 10.1038/s41419-021-03713-9

**Published:** 2021-05-01

**Authors:** Germana Zaccagnini, Simona Greco, Marialucia Longo, Biagina Maimone, Christine Voellenkle, Paola Fuschi, Matteo Carrara, Pasquale Creo, Davide Maselli, Mario Tirone, Massimiliano Mazzone, Carlo Gaetano, Gaia Spinetti, Fabio Martelli

**Affiliations:** 1 Laboratory of Molecular Cardiology, IRCCS Policlinico San Donato, 20097 San Donato Milanese, Milan, Italy; 2 Laboratory of Stem Cells for Tissue Engineering, IRCCS Policlinico San Donato, 20097 San Donato Milanese, Milan, Italy; 3 King’s College London, School of Cardiovascular Medicine and Sciences, BHF Center of Research Excellence, London, UK; 4 Division of Genetics and Cell Biology, Chromatin Dynamics Unit, San Raffaele University, 20132 Milan, Italy; 5 Laboratory of Tumor Inflammation and Angiogenesis, Center for Cancer Biology (CCB), VIB, and Department of Oncology, KU Leuven, 3000 Leuven, Belgium; 6 Laboratorio di Epigenetica, Istituti Clinici Scientifici Maugeri IRCCS, via Maugeri 4, 27100 Pavia, Italy; 7 Laboratory of Cardiovascular Research, IRCCS MultiMedica, 20138 Milan, Italy

**Keywords:** Cardiovascular diseases, Inflammation

## Abstract

Hypoxia-induced miR-210 is a crucial component of the tissue response to ischemia, stimulating angiogenesis and improving tissue regeneration. Previous analysis of miR-210 impact on the transcriptome in a mouse model of hindlimb ischemia showed that miR-210 regulated not only vascular regeneration functions, but also inflammation. To investigate this event, doxycycline-inducible miR-210 transgenic mice (Tg-210) and anti-miR-210 LNA-oligonucleotides were used. It was found that global miR-210 expression decreased inflammatory cells density and macrophages accumulation in the ischemic tissue. To dissect the underpinning cell mechanisms, Tg-210 mice were used in bone marrow (BM) transplantation experiments and chimeric mice underwent hindlimb ischemia. MiR-210 overexpression in the ischemic tissue was sufficient to increase capillary density and tissue repair, and to reduce inflammation in the presence of Wt-BM infiltrating cells. Conversely, when Tg-210-BM cells migrated in a Wt ischemic tissue, dysfunctional angiogenesis, inflammation, and impaired tissue repair, accompanied by fibrosis were observed. The fibrotic regions were positive for α-SMA, Vimentin, and Collagen V fibrotic markers and for phospho-Smad3, highlighting the activation of TGF-β1 pathway. Identification of Tg-210 cells by in situ hybridization showed that BM-derived cells contributed directly to fibrotic areas, where macrophages co-expressing fibrotic markers were observed. Cell cultures of Tg-210 BM-derived macrophages exhibited a pro-fibrotic phenotype and were enriched with myofibroblast-like cells, which expressed canonical fibrosis markers. Interestingly, inhibitors of TGF-β type-1-receptor completely abrogated this pro-fibrotic phenotype. In conclusion, a context-dependent regulation by miR-210 of the inflammatory response was identified. miR-210 expression in infiltrating macrophages is associated to improved angiogenesis and tissue repair when the ischemic recipient tissue also expresses high levels of miR-210. Conversely, when infiltrating an ischemic tissue with mismatched miR-210 levels, macrophages expressing high miR-210 levels display a pro-fibrotic phenotype, leading to impaired tissue repair, fibrosis, and dysfunctional angiogenesis.

## Introduction

Critical limb ischemia (CLI) is a pathological condition in which blood flow is insufficient to meet the metabolic demand. This pathology is characterized by severe functional consequences, difficult clinical management, and reduced life expectancy^1^. CLI is estimated to affect more than 200 million people worldwide^2^ and it is mostly caused by stenosis, embolism, or thrombosis involving the arteries supplying the leg^3^.

The restoration of tissue homeostasis after an ischemic insult requires the activation of two main processes: inflammation and angiogenesis. Indeed, after ischemia, infiltrating bone marrow (BM) derived inflammatory cells play a crucial role in removing damaged and dead cells, and in promoting tissue repair, while endothelial cells, pericytes, and smooth muscle cells participate in rebuild the vascular network and re-establish oxygen supply. Interestingly, blood vessel formation and immune response exhibit reciprocal regulation^4^. Although a moderate inflammation may stimulate blood vessel formation and restore local homeostasis, a chronic response may lead to permanent dysfunction and tissue fibrosis^5^.

Among the plethora of cell types involved in the tissue response to damage, macrophages have been shown to exert crucial regulatory functions in each step of healing and fibrosis. Indeed, macrophages are highly plastic cells and can assume either a pro-inflammatory or an anti-inflammatory, pro-repair phenotype in response to growth factors, cytokines, and other mediators released in the local tissue microenvironment^5,6^.

TGF-β plays an important role in muscle and cardiac fibrosis^7–9^. In patients affected by peripheral artery disease, TGF-β1 expression progressively increases with advancing severity and positively correlates with collagen density^8^. In addition, the progressive fibrosis observed in CLI patients is, at least in part, caused by elevated TGF-β1 production in smooth muscle cells of microvessels, in response to tissue hypoxia^10^.

miR-210 is a microRNAs expressed in response to hypoxia and ischemia and modulates several target genes involved in these processes^11^. Indeed, miR-210 has been found upregulated in different ischemic diseases, such as hindlimb ischemia in mice^12^, brain transient focal ischemia in rats^13^, ischemic wounds in mice^14^, myocardial infarction in humans^15^, and in heart failure of diabetic patients affected by dilated ischemic cardiomyopathy^16^.

miR-210 modulates several processes activated in hypoxia response by targeting specific mRNAs. In particular, it represses mitochondrial metabolism, promoting the shift from mitochondrial respiration to glycolysis^17^, inhibits apoptosis^12,18^, supports stem-cell survival^19^, and stimulates angiogenesis^18,20^. Accordingly, in hindlimb ischemia, we previously demonstrated that miR-210 inhibition increased ROS-mediated tissue damage due to insufficient downregulation of oxidative metabolism^12^ and, on the contrary, miR-210 over-expression in an inducible transgenic mouse model, protected from acute ischemic damage^21^ and promoted angiogenesis^20^. Nevertheless, miR-210 function is complex and might be highly context- and time-dependent. Indeed, as for all microRNAs, the function of miR-210 depends on the target mRNAs expressed by each cell type and, as a consequence, the biological effect of its expression can vary according to the context.

In a previous work, we evaluated the miR-210 impact on the transcriptome^20^. Our results indicated that miR-210 regulates gene categories related not only to angiogenesis, but also to stress response, immune response, and inflammation. The goal of this study was to investigate how miR-210 regulation of the inflammatory system affects the angiogenic and tissue regeneration processes upon acute ischemia.

## Results

### miR-210 over-expression attenuates the inflammatory response after hindlimb ischemia

Previous transcriptomic analysis indicated that miR-210 inhibition in a mouse model of hindlimb ischemia, modulates gene targets related to immune response and inflammation^20^. To study miR-210 role in the inflammatory response after acute ischemia, miR-210 levels were modulated after femoral artery dissection, either positively or negatively, using an established experimental strategy (Fig. S1). Since we previously demonstrated that inhibiting miR-210 before ischemia increases ischemic tissue damage^12^, miR-210 levels were modulated after ischemia, when the maximal vascular damage phase was over and the regeneration phase had begun, starting from similar levels of tissue damage in each ischemic group^20^.

**Fig. 1:**
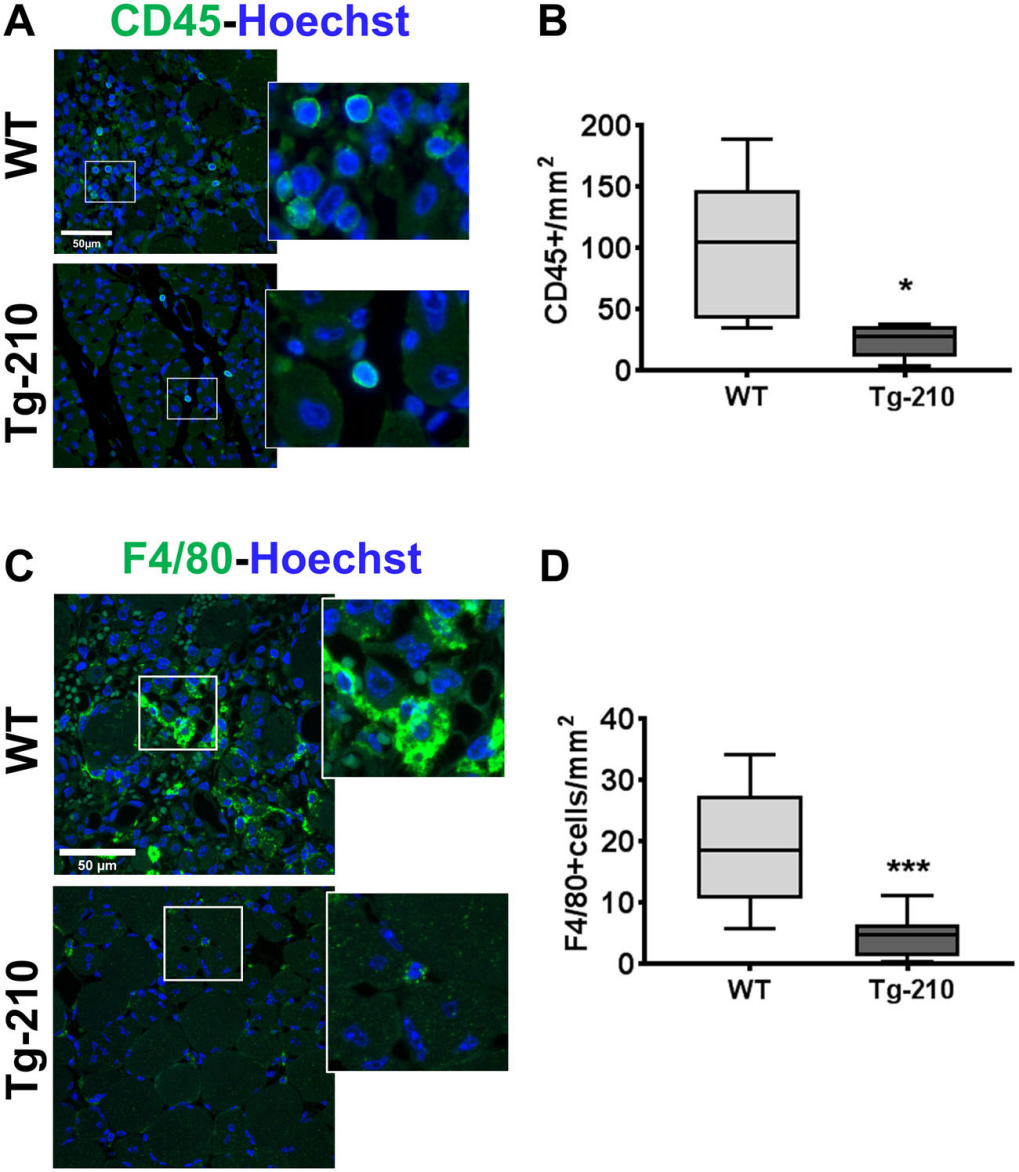
Ubiquitous miR-210 over-expression attenuates the inflammatory response after hindlimb ischemia. **A** Representative immunofluorescence staining for CD45 (green), a common leukocyte marker expressed in all nucleated hematopoietic cells, of gastrocnemius muscle sections of Wt and Tg-210 mice, 7 days after ischemia. Nuclei were stained by Hoechst (blue). Images are presented as merge. Magnification ×400. Calibration bar 50 µm. **B** Quantification of CD45-positive cells/mm^2^ (*n* = 5, test *T* *P < 0.03). **C** Representative immunofluorescence staining for F4/80 (green), a pan-macrophage marker, in gastrocnemius muscle sections of Wt, and Tg-210 mice, 7 days after ischemia. Nuclei were stained by Hoechst (Blue). Images are presented as merge. Magnification 63 × 0.5. Calibration bar 50 µm. **D** Quantification of F4/80-positive macrophages/mm^2^ (*n* = 6–7, **P < 0.009). In both **A**, **C** insets show magnification of the indicated areas.

First, we validated a subset of genes identified by microarray analysis^20^ belonging to the immune response and inflammation categories, confirming their regulation in the ischemic muscle by qPCR (Fig. S2). Next, we investigated how the modulation of miR-210 affects the inflammatory response in ischemic tissues. To this aim, the presence of inflammatory cells was evaluated in ischemic gastrocnemius muscles by immunofluorescence staining for pan-leukocyte (Fig. 1A) and pan-macrophage markers (Fig. 1C). Leukocytes infiltration was significantly decreased when miR-210 was induced in Tg-210 ischemic mice (Fig. 1B) and in particular macrophage density was significantly reduced (Fig. 1D). Conversely, macrophage density was increased after miR-210 blocking in ANTI-210 treated mice (Fig. S3). Taken together, these data indicate that ubiquitous miR-210 expression attenuates the inflammatory response after hindlimb ischemia.

### Wt mice transplanted with Tg-210 BM cells display impaired tissue repair, dysfunctional angiogenesis, and decreased calf perfusion after ischemia

Considering the positive role played by miR-210 in angiogenesis^18,20^, we wondered whether a local microenvironment enriched for miR-210 is sufficient in promoting angiogenesis after injury, or, conversely, it is miR-210 level in BM-derived inflammatory cells to be crucial for this process. BM-transplantation experiments were performed to answer this question, using CD45 allelic variants (CD45.1 and CD45.2) to distinguish transplanted hematopoietic-lineage cells in chimeric mice^22^. Peripheral blood flow-cytometry showed that Tg-210 mice and Wt littermates expressed the CD45.2 allele (Fig. S4). Therefore, CD45.2-positive BM cells from Tg-210 donor mice were transplanted in CD45.1-positive Wt recipient mice (BM-Tg210/R-wt) (Fig S5A); alternatively, BM-cells from CD45.1-positive Wt donor mice were transplanted in CD45.2-positive Tg-210 recipient mice (BM-wt/R-Tg210) (Fig. S5B). Two additional control groups were generated, in order to monitor unexpected side effects of the transplantation procedure: BM-wt/R-wt and BM-Tg210/R-Tg210 (Fig. S5C). Only mice with a virtually complete substitution (>98%) of the BM-cells were analyzed, allowing to investigate the biological effect of BM-derived cells overexpressing miR-210 in a Wt background and vice versa. Then, reconstituted mice underwent hindlimb ischemia and four days later miR-210 was induced. At day 14, calf perfusion was measured and mice were sacrificed for histological evaluations of the gastrocnemius muscle (Fig. S5).

**Fig. 2:**
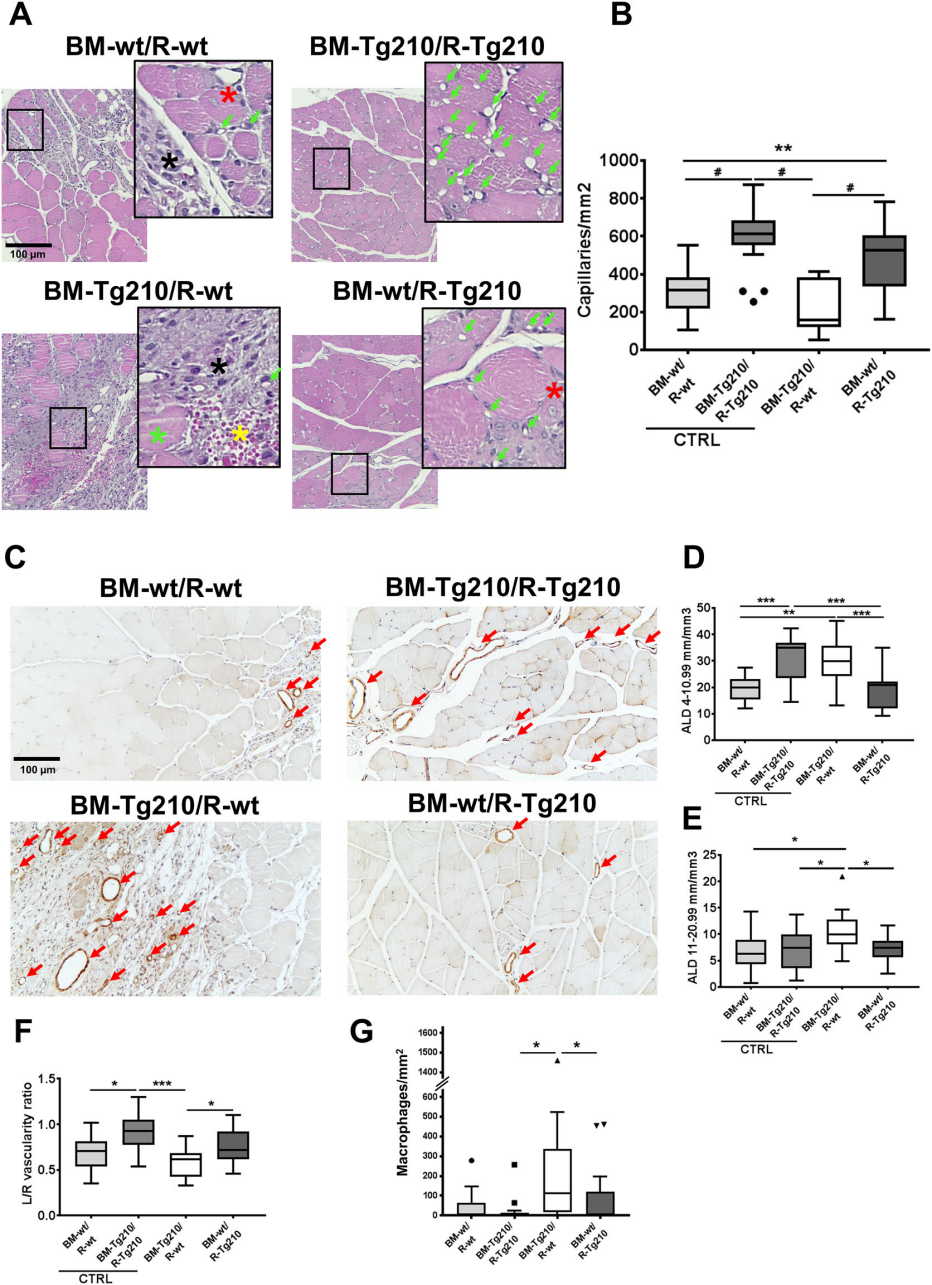
BM-Tg210/R-wt muscles display impaired tissue repair, dysfunctional angiogenesis, and decreased calf perfusion after ischemia. **A** Representative H/E stained sections of ischemic gastrocnemius muscles of BM-wt/R-wt, BM-Tg210/R-Tg210, BM-Tg210/R-wt, and BM-wt/R-Tg210 mice. Magnification ×200. Calibration bar 100 µm. Insets show magnifications of the indicated areas. Green arrows indicate capillaries; black asterisks indicate areas of inflammation, green asterisks indicate degenerating myofibers, red asterisks indicate regenerating myofibers (i.e., small myofibers with central nucleus) and yellow asterisks indicate extravasated erythrocytes. **B** Capillary density quantification (*n* = 16–18, Anova multiple comparison **P < 0.002, ^#^*P* < 0.0001). **C** Representative α-SMA immunohistochemistry of ischemic gastrocnemius muscles of BM-wt/R-wt, BM-Tg210/R-Tg210, BM-Tg210/R-wt, and BM-wt/R-Tg210 mice. Red arrows indicate arterioles. Magnification ×200, calibration bar 100 µm. **D**, **E** Arteriolar length density (ALD) quantification. Arterioles were classified on the basis of the minimum internal diameter as small (4–10.99 μm in **D**) and medium (11–20.99 μm in **E**) (*n* = 16–18 Anova multiple comparison *P < 0.05; **P < 0.002; ***P < 0.0007). **F** Calf perfusion was monitored by ultrasound in power Doppler mode and expressed as vascularity ratio of ischemic/non-ischemic limbs (*n* = 16–18, Anova multiple comparison *P < 0.05; ***P < 0.0001). **G** Macrophage density quantification (*N* = 16–18, Kruskal–Wallis test, multiple comparison *P* < 0.04).

First, the morphology of ischemic muscles was analyzed in H/E stained sections (Fig. 2A). Muscles of both control groups and of BM-wt/R-Tg210 group displayed an advanced tissue-regeneration process. Conversely, extensive myofiber damage, extravasated erythrocytes, inflammation, and few or absent regenerating myofibers were observed in almost all BM-Tg210/R-wt mice. To better clarify this finding, we further evaluated the angiogenic response, the presence of macrophages, and tissue fibrosis.

Capillaries density was significantly higher in BM-Tg210/R-Tg210 group compared to both BM-wt/R-wt and BM-Tg210/R-wt groups (Fig. 2B), but was similar to that observed in BM-wt/R-Tg210 group, highlighting the importance of miR-210 tissue levels for the pro-angiogenic function of miR-210. Remarkably, BM-Tg210/R-wt showed significantly lower capillary density compared not only to BM-Tg210/R-Tg210, but also to BM-wt/R-Tg210 mice. Arteriolar length density (ALD) of small-size (i.e., 4–10.99 µm range) was significantly higher in the BM-Tg210/R-Tg210 group compared to both BM-wt/R-wt and BM-wt/R-Tg210 groups (Fig. 2C). Surprisingly, BM-Tg210/R-wt mice displayed significantly higher ALD compared to both BM-wt/R-wt and BM-wt/R-Tg210 mice (Fig. 2D). This ALD increase observed in BM-Tg210/R-wt mice was even more pronounced when larger arterioles (i.e., 11–20.99 µm range) were analyzed, indicating also an increase in arteriolar size (Fig. 2E). However, this ALD increase was not coupled to an improvement of calf perfusion. Indeed, significantly lower vascularity ratio of ischemic/non-ischemic limbs was observed in BM-Tg210/R-wt ischemic muscles compared to both BM-Tg210/R-Tg210 and BM-wt/R-Tg210 (Fig. 2F).

Additionally, total macrophage density was significantly increased in BM-Tg210/R-wt ischemic muscles compared to BM-wt/R-Tg210 (Fig. 2G).

Taken together, these results show that BM-Tg210/R-wt chimeric mice display impaired tissue repair, dysfunctional angiogenesis, and increased inflammation after ischemia.

### BM-Tg210/R-wt ischemic muscles display tissue fibrosis

**Fig. 3:**
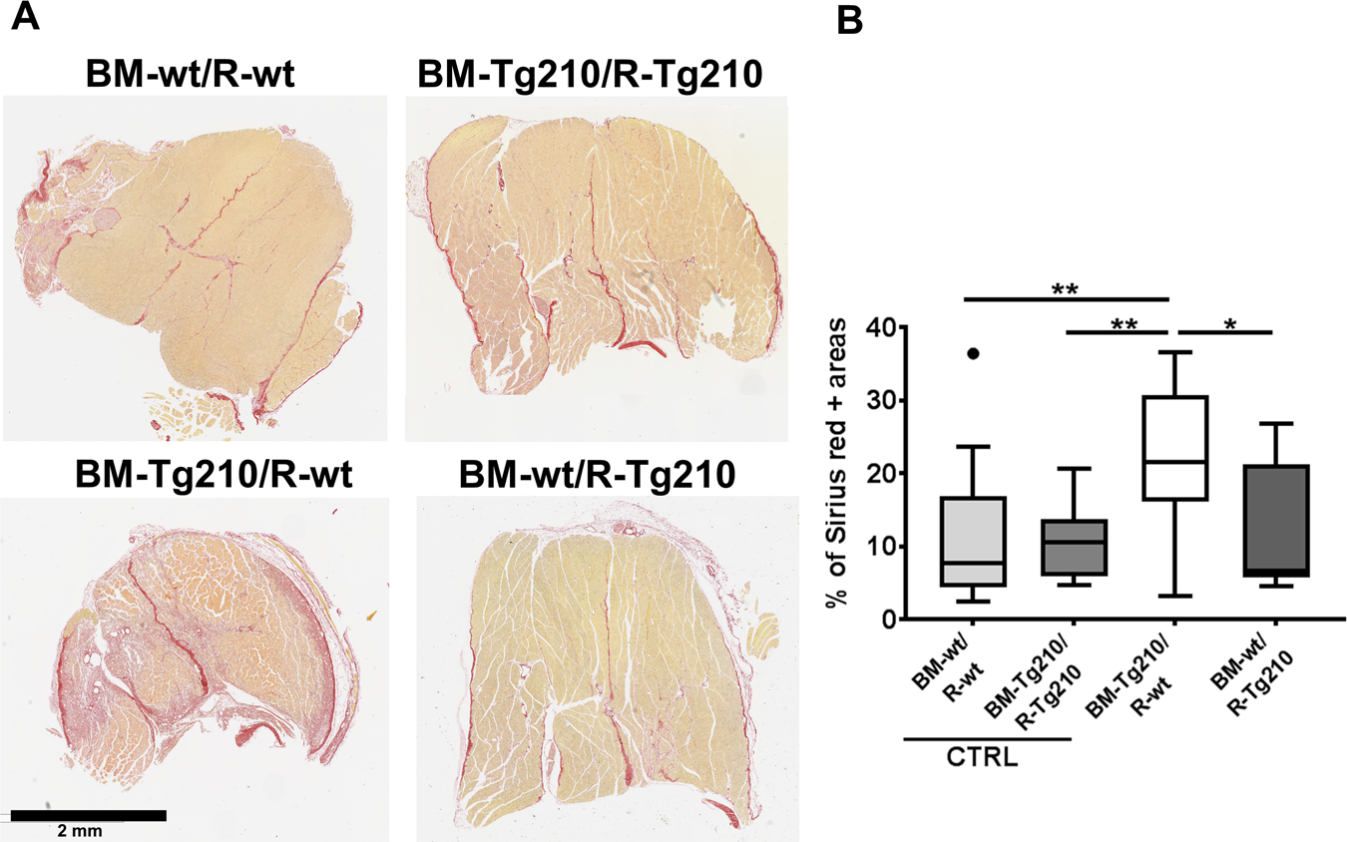
BM-Tg210/R-wt ischemic muscles display tissue fibrosis after ischemia. **A** Representative images of Sirius red stained sections of ischemic gastrocnemius muscles of BM-wt/R-wt, BM-Tg210/R-Tg210, BM-Tg210/R-wt, and BM-wt/R-Tg210 mice. Collagen fibers were stained in red. Calibration bar 2 mm. **B** Percentage of Sirius red positive areas (*n* = 16–18, Anova multiple comparison *P < 0.01; **P < 0.005).

Since BM-Tg210/R-wt mice show extensive areas of tissue damage in H/E stained sections, the presence of tissue fibrosis was assessed by Sirius red staining of collagen fibers (Fig. 3A). The areas of collagen deposition were significantly increased in ischemic muscles of BM-Tg210/R-wt mice compared with other groups, suggesting the presence of fibrosis (Fig. 3B). This finding was specific of the BM-Tg210/R-wt group, as all the other did not display any increase in collagen deposition compared to the non-ischemic contralateral limb (about 10% in all groups).

To further confirm this result, the presence of myofibroblasts was investigated in the ischemic tissue by staining for α-SMA, Vimentin, and Collagen V on serial sections of ischemic muscles (Fig. S6). We found that large areas of BM-Tg210/R-wt ischemic muscles co-expressed these three markers, suggesting the presence of myofibroblasts. As expected, the other groups of chimeric mice expressed α-SMA and Vimentin mostly in the arteries and myofibroblasts were virtually absent.

Taken together, these results show that BM-Tg210/R-wt mice display an impaired tissue repairing of the ischemic damage and develop marked fibrosis.

### TGF-β1 pathway activation in BM-Tg210/R-wt ischemic muscles

**Fig. 4:**
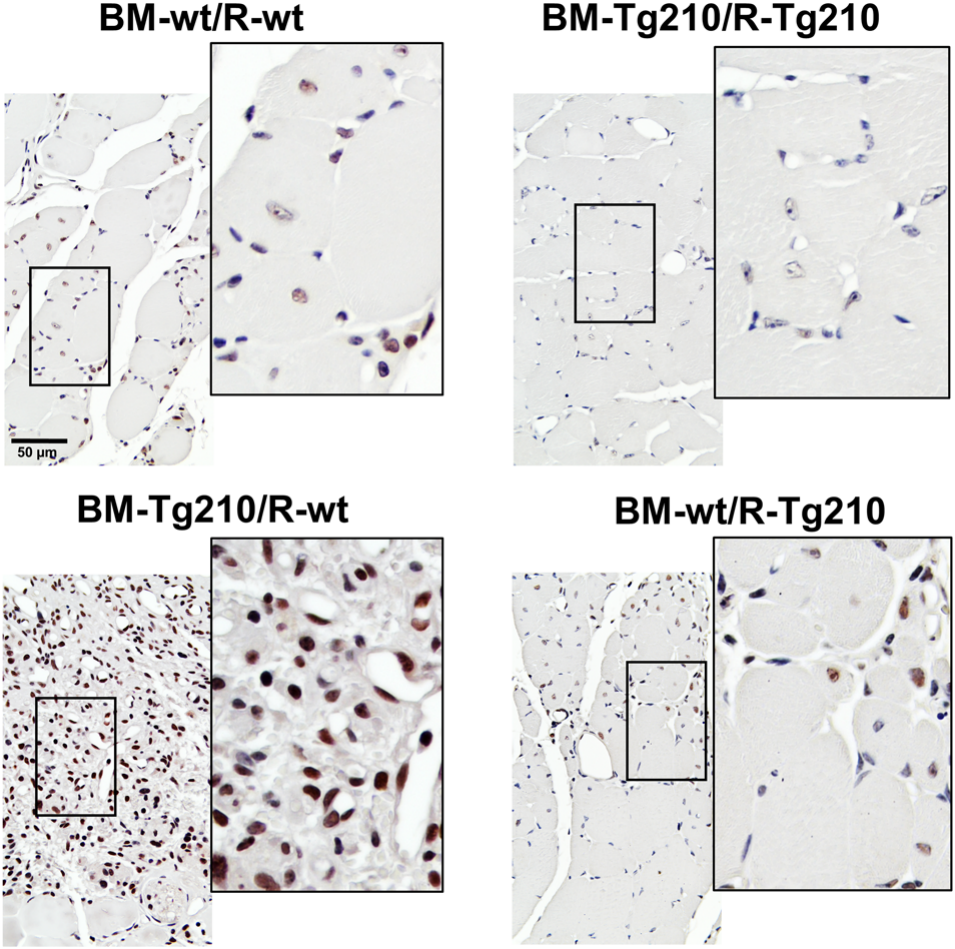
Activation of TGF-β1-Smad3-P signaling pathway in ischemic BM-Tg210/R-wt gastrocnemius muscles. Representative images of anti-phospho-Smad3 IHC of ischemic gastrocnemius muscle sections of BM-wt/R-wt, BM-Tg210-R-Tg210, BM-Tg210-R-wt, and BM-wt/R-210 chimeric mice. Positive nuclei are stained in brown/black (Smad3-P + hematoxylin), negative nuclei are stained in blue (hematoxylin alone). Magnification ×400, calibration bar 50 µm. Insets show magnifications of the indicated areas.

Since TGF-β1 plays a pivotal role in collagen accumulation and fibrosis in several diseases^5,7^, the activation of TGFβ-1 pathway was assessed by immunohistochemical staining for phospho-Smad3 (Smad3-P), a key intracellular mediator of TGFβ-1^23,24^. In BM-Tg210/R-wt mice, a strong Smad3-P staining was evident in almost all of the nuclei of ischemic muscles (Fig. 4). Conversely, in the other chimeric mice (BM-wt/R-wt, BM-Tg210/R-Tg210, BM-wt/R-Tg210) Smad3-P-positive nuclei were detected only in the regenerating myofibers or in interstitial cells of small areas of residual inflammation, and no staining was present in the healthy regions of the muscles. Similarly, only few positive nuclei were found in the ischemic muscles of non-transplanted mice (Fig. S7). Taken together these results suggest a pervasive activation of the TGF-β1-Smad3-P pathway only in BM-Tg210/R-wt ischemic muscles.

### Macrophages of BM-Tg210/R-wt mice display a pro-fibrotic phenotype

The role of inflammatory infiltrating cells in the fibrosis observed in BM-Tg210/R-wt muscles was further investigated. In this case, only BM-wt/R-Tg210 and BM-Tg210/R-wt groups were analyzed, since the two control groups, BM-wt/R-wt and BM-Tg210/R-Tg210, showed angiogenic response and tissue repair very similar to those observed in non-chimeric Wt and Tg-210 mice, respectively.

**Fig. 5:**
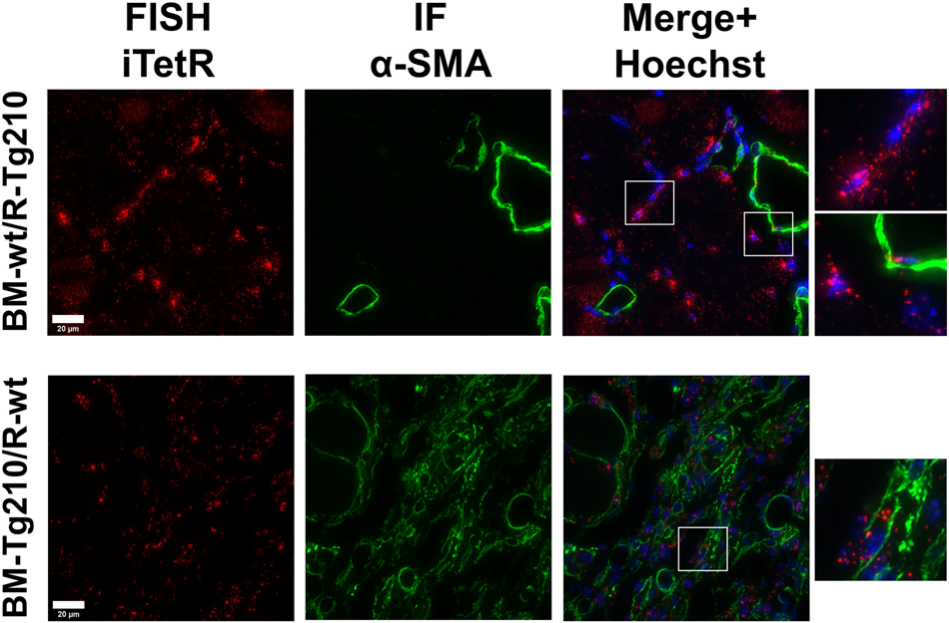
α-SMA expression by BM-derived Tg-210 inflammatory cells. Representative images of double immunofluorescence staining for iTetR in situ hybridization (left, red), α-SMA (center, green) and merge (right) of ischemic gastrocnemius muscles sections of BM-wt/R-Tg210 mice and BM-Tg210/R-wt. Nuclei were stained by Hoechst (Blue). iTetR mRNA displays both nuclear and perinuclear staining. Representative images are presented as Maxprojections. Magnification 63 × 0.5, calibration bar 20 µm.

In particular, we wondered whether BM-derived inflammatory cells could be direct contributors of the muscle fibrosis areas identified by the expression of α-SMA protein^25^. Thus, BM-derived Tg-210 cells were identified in chimeric mice by in situ hybridization (ISH) assay, using a probe for the exogenous mRNA of iTetR, which is expressed only in cells derived from Tg-210 mice. Fig. S8 shows that, as expected, Wt muscles did not express iTetR mRNA, while Tg-210 mice expressed iTetR mRNA in all cell types, with both nuclear and perinuclear localization, confirming the specificity of the adopted assay. As additional control, ischemic muscle of BM-wt/R-Tg210 mice were tested (Fig. 5, top). As expected, in these chimeric mice, double-positive α-SMA/iTetR cells were only observed in the arteries.

In BM-Tg210/R-wt ischemic muscle (Fig. 5, bottom panels), cells positive for both iTetR and α-SMA were observed throughout the sections, highlighting the presence of cells of BM-origin overexpressing miR-210 and α-SMA in the fibrotic areas. This finding was confirmed by the identification of CD45/α-SMA (Fig. S9A) and CD45/Collagen V double-positive cells (Fig. S9B) in BM-Tg210/R-wt ischemic muscles, but not in BM-wt/R-Tg210 samples.

**Fig. 6:**
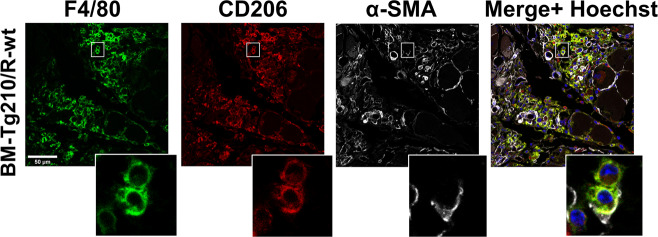
BM-Tg210/R-wt ischemic muscles display α-SMA expressing macrophages. Representative images of triple immunofluorescence staining for F4/80 (green), CD206 (red), α-SMA (white) of ischemic gastrocnemius muscles sections of BM-wt/R-Tg210 and BM-Tg210/R-wt mice. The merge include Hoechst (blue) nuclear staining. Representative images are presented as Maxprojections. Magnification 63 × 0.5, calibration bar 50 µm. Insets show magnifications of the indicated areas.

To further characterize these BM-derived cells, muscle sections of chimeric mice were stained for F4/80 (a pan-macrophage marker), CD206 (marker of M2-polarized macrophages) and α-SMA. F4/80/CD206 triple-positive macrophages co-expressing α-SMA were reproducibly observed in BM-Tg210/R-wt ischemic muscles (Fig. 6) and only occasionally in BM-wt/R-Tg210 mice (Fig. 6 and Fig. S10).

Taken together these results show that miR-210-expressing macrophages infiltrating in Wt ischemic muscles can display a myofibroblast-like, pro-fibrotic phenotype.

### Cell cultures of Tg-210 BM-derived macrophages exhibit a pro-fibrotic phenotype

To clarify the role of miR-210 over-expressing macrophages in fibrosis, BM cells were isolated from Wt or Tg-210 donor mice and cultured in selecting conditions allowing macrophages differentiation (Fig. S11).

After 7 days of differentiation in non-adhering dishes, cells were transferred in glass chamber slides. One day later (day 7 + 1), macrophage cultures where characterized: more than 99.5% of the cells were positive for CD68 (Fig. S12A and B) and no cells expressing α-SMA or Collagen I were detected (Fig. S12D), indicating highly pure macrophage cultures. Cells were also positive for CD206, indicating a polarization toward a M2-like phenotype (Fig. S12C).

**Fig. 7:**
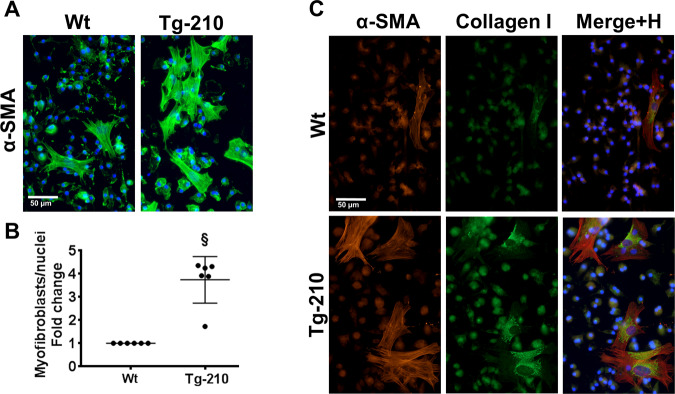
Cell culture of Tg-210 BM-derived macrophages exhibit a pro-fibrotic phenotype. **A** Representative immunofluorescence staining for α-SMA (green) of BM-derived macrophages at day 7 + 7 of culture. Images are presented as merge, nuclei were stained by Hoechst (blue). Magnification ×400, calibration bar 50 µm. **B** Quantification of myofibroblasts/total nuclei, represented as fold change versus Wt (*n* = 4, §*P* < 0.0001). **C** Representative images of double immunofluorescence staining for α-SMA and Collagen I of BM-derived macrophages at day 7 + 7 of culture. The merges include the nuclear staining by Hoechst (H) (blue). Magnification ×400, calibration bar 50 µm.

Next, we analyzed the pro-fibrotic phenotype of macrophage cultures at day 7 + 7. IF staining for α-SMA showed a significantly higher density of myofibroblast-like cells in Tg-210 macrophages cultures than in Wt (Fig. 7A, B). These cells co-expressed α-SMA and Collagen I (Fig. 7C), confirming the myofibroblast phenotype. qPCR analysis of fibrosis markers confirmed significantly higher levels of ACTA-2, Coll1A1, Coll3A1, MYH10 in Tg-210 macrophage cultures compared to Wt (Fig. S13).

Given the TGF-β-pathway activation observed in BM-Tg210/R-wt ischemic muscles, the impact of TGF-β type 1-receptor inhibition was assessed. Using two different inhibitors (A-83-01 or SB431542), we found that TGF-β1-pathway inhibition completely abrogated the generation of α-SMA positive cells in Tg-210 macrophage cultures (Fig. S14).

Taken together these results demonstrate that macrophage cultures over-expressing miR-210 display a pro-fibrotic phenotype.

## Discussion

There is mounting evidence of an immune-modulatory functions of miR-210, as it regulates T lymphocyte subsets in psoriasis^26^, inhibits the production of pro-inflammatory cytokines in LPS-stimulated macrophages^27^, potentiates the tumor-promoting effects of myeloid-derived suppressor cell^28^ and inhibits the pro-inflammatory immune responses of lehismania-infected macrophages^29^.

Here, we investigated miR-210 function in the context of the inflammatory response to acute ischemia. First, miR-210 levels were modulated ubiquitously. The results showed that miR-210 attenuates the pro-inflammatory response, promoting a pro-repair response. These findings are in accordance with a previous study in which we demonstrated that miR-210 blockade before ischemia increases tissue inflammation^21^.

According to the organ or tissue, physiological or pathological conditions and the timing, the same miRNA can modulate different gene pathways, potentially leading to opposing effects in different contexts^30,31^. Considering the complex array of miR-210 functions^11^, we hypothesized this was true also in the context of the ischemia response. Indeed, in a mouse model of ischemic stroke, miR-210 inhibition suppresses the pro-inflammatory response and reduces acute brain injury^32^. Thus, we investigated if a miR-210-enriched tissue, infiltrated by Wt inflammatory cells, is sufficient in stimulating angiogenesis after injury, or, alternatively, it is miR-210 level in BM-derived inflammatory cells alone, to act as driver for this process. To answer this question we generated chimeric mice, using a bone marrow transplantation approach, to investigate both mechanisms with a single experimental approach, allowing a direct comparison of the different experimental groups.

In keeping with our previous observations showing that ubiquitous miR-210 over-expression in Tg-210 ischemic mice improves the angiogenic response and perfusion recovery^20^, efficient calf perfusion and angiogenesis were observed in BM-wt/R-Tg210 mice. This indicates that a miR-210-expressing tissue is sufficient to stimulate vascular and tissue regeneration. Quite surprisingly, miR-210 over-expression in BM-derived infiltrating cells was not simply ineffective in stimulating angiogenesis and tissue repair; conversely, ischemic muscles of BM-Tg210/R-wt mice displayed decreased calf perfusion, dysfunctional angiogenesis, and impaired tissue repair, associated with increased macrophage density and fibrosis.

Dysfunctional angiogenesis in BM-Tg210/R-wt mice was characterized by impaired capillary density and calf perfusion as well as extravasated erythrocytes, associated to an apparently paradoxical ALD increase. The reason of this phenotype is unclear. Nevertheless, there is evidence that macrophages may promote the activation of smooth muscle cells (SMCs) by releasing PDGF^33^ and IL-6^34^. In this respect, we found that a portion of iTetR-positive BM-derived inflammatory cells were adherent to the α-SMA-positive SMCs of the arteries in BM-210/R-wt ischemic muscles (data not shown). Moreover, several studies highlighted an interplay between SMCs and macrophages in atherosclerosis^35^, and miR-210 enhances fibrous cap stability in advanced atherosclerotic lesions by inhibiting SMCs apoptosis^36^.

Remarkably, fibrosis was observed only in BM-Tg210/R-wt ischemic muscles and not in the other chimeric groups. In addition, in our previous studies, under no circumstance tissue fibrosis was observed after hindlimb ischemia in mice upon ubiquitous modulation of miR-210^12,20,21^. Thus, we further investigated this feature of miR-210 over-expression in infiltrating cells. Interestingly, evidence of TGF-β1/Smad3 signaling was found in almost all the nuclei of BM-Tg210/R-wt ischemic muscles. In the other chimeric groups, we found only weak signals of Smad3-P, mostly located in the nuclei of regenerating myofibers, in keeping with the role played by TGF-β1 and Myostatin in skeletal muscle regeneration after injury^23,24^. There is evidence that miR-210 could modulate TGF-β family pathway. Indeed, among its direct targets, there are both positive and negative effectors of TGF-β pathway^37–40^. Since the function of TGF-β family members and effectors is highly cell and tissue specific, their role in this specific context can be further investigated in future studies.

These results prompted us to further study miR-210 role in the regulation of fibrosis. In BM-Tg210/R-wt ischemic muscles, a population of BM-derived infiltrating cells, co-expressing α-SMA and/or Collagen V was identified, indicating a direct contribution to tissue fibrosis. Additional analysis showed that these BM-derived α-SMA-positive cells were, at least in part, M2 polarized macrophages, highlighting a pro-fibrotic phenotype. These results are in keeping with a previous work demonstrating that macrophages directly contribute collagen to the forming post-injury scar in both zebrafish and mouse models of cardiac injury^41^ and with the results obtained in a mouse model of wound healing, showing that two-thirds of all granulation tissue fibroblasts are derived from myeloid cells and are likely to be wound macrophages^42^. Accordingly, in kidneys, TGF-β1/Smad3-P signaling regulates the transition of BM-derived macrophages into myofibroblasts, leading to inflammation and tissue fibrosis^43,44^ and CD206-positive M2-macrophages are strongly associated with renal fibrosis, both in humans and in experimental diseases^44^. One may also speculate that the observed pro-fibrotic cells could be, at least in part, fibrocytes, as they have been implicated in the pathogenesis of multiple diseases such as idiopathic pulmonary fibrosis or corneal fibrosis^25,45^, and in tissue healing in experimental models of muscle injury and muscular dystrophy^46^. Fibrocytes appear to differentiate from peripheral blood monocytes^47^, bear characteristics of both fibroblasts and monocytes and are able to differentiate in myofibroblast-like cells increasing their expression of TGFβ and endothelin^25^. Interestingly, fibrocytes might play a crucial role in organ fibrosis through the CXC chemokine receptor 4 (CXCR4)/stromal‑derived factor‑1 (SDF‑1 α/CXCL12) axis^48^ and we previously demonstrated that miR-210 over-expression enhance circulating pro-angiogenic cell migration toward SDF-1 α/CXCL12^49^.

The pro-fibrotic role of miR-210 identified in vivo was also supported by in vitro experiments, as myofibroblast-like, co-expressing fibrosis markers such as α-SMA and Collagen I, developed preferentially in cultures of BM-derived macrophages overexpressing miR-210. Significantly, this pro-fibrotic phenotype was completely abrogated when TGF-β1 pathway was inhibited^7–9^. Since both macrophages and fibrocytes originate from monocytes, co-express the majority of the markers and could be differentiated and cultured in similar condition^47^, this cell type may be present in BM-derived macrophage populations cultured in the adopted experimental conditions. This raises the possibility that the observed myofibroblast-like cells are indeed derived from fibrocytes, or that fibrocytes exert a paracrine pro-fibrotic effect on macrophages^45^. Although at day 7 + 1 the majority of BM-derived cells are positive for CD68 and negative for αSMA or Collagen I, we cannot formally exclude the presence of contaminant cells of mesenchymal origin. Even in this case, the results would indicate a strong paracrine pro-fibrotic stimulus present in miR-210 overexpressing cultures.

Other studies also identified a miR-210 role in macrophage-mediated physio-pathological mechanisms. Indeed, an HIF-1α-dependent miR-210 upregulation in monocytes and macrophages has been observed in response to pathogen interaction in different mouse infection models, and miR-210 inhibition/deletion is beneficial in bacteremia, endotoxemia, sepsis and parasitosis^50^. Specifically, miR-210 induction in macrophages, by repressing mitochondrial respiration and promoting glycolysis, leads to a switch towards a pro-inflammatory, M1 phenotype. Interestingly miR-210 level in circulating monocytes of cancer patients, positively correlates with the incidence of sepsis and increased serum levels of miR-210, derived from monocyte/macrophage, is associated with sepsis mortality^51^. Moreover, an HIF-1α-dependent miR-210 upregulation has also been observed in myeloid-derived suppressor cells, significantly contributing to the malignant character conferred by hypoxic tumor microenvironment^28^.

In conclusion, miR-210 over-expression, in macrophages infiltrating in an ischemic muscle, is associated to efficient angiogenesis and tissue repair, when the damaged recipient tissue over-expresses miR-210 too. On the contrary, in a milieu expressing physiological levels of miR-210, over-expression of miR-210 in infiltrating macrophages causes impaired tissue repair, fibrosis, and dysfunctional angiogenesis (Fig. S15).

Taken together our findings confirm the pro-angiogenic, pro-regenerative, anti-inflammatory role of mir-210 after hindlimb ischemia. Nevertheless, our findings highlight that, given the high pleiotropy of each microRNA, miR-210 function needs to be tested in a variety of different physiopathological contexts. This accurate preclinical work is necessary to fully understand its function, before to consider it for translational applications in CLI patients.

## Materials and methods

### Mouse models

For ANTI-210 experiments, 8–12 weeks old C57BL/6N male mice (Charles River Laboratories, Calco (MI), Italy) were used (*N* = 4). For transgenic mouse experiments, 8–12 weeks old doxycycline inducible transgenic C57BL/6NTac-*Gt(ROSA)26Sor*^*tm3720(Mir210)Tac*^ (Tg-210, Taconic Artemis, Germany) male mice and Wild Type littermate (Wt) were used (*N* = 6–7). The generation of Tg-210 mice has been previously described in detail^21^. Briefly, the miR-210 coding region was inserted into the ROSA26 locus by using a targeting strategy that allows doxycycline inducible overexpression of miR-210.

All mice were housed in groups of three to five at 22° ± 2 °C using a 12 h light-12 h dark cycle.

Animals were fed with normal chow diet (SDS, irradiate VRF1) until doxycycline administration. For miR-210 induction, Wt and Tg-210 mice were fed with pellets of food containing Doxycycline (NFM18 diet added with doxycycline hyclate 2000 mg/kg, Mucedola, Settimo Milanese (MI), Italy) (Fig. S1A). Doxycycline was administrated to Wt littermate too, in order to exclude side effects of the drug. The effectiveness of miR-210 induction was assessed by qPCR on quadriceps femoris muscles or liver samples of each mouse analyzed.

Before all surgical and perfusion procedures, mice were anesthetized with an intraperitoneal injection of 10 mg/kg xylazine (Intervet Farmaceutici, Pesciera Borromeo (MI), Italy) and 100 mg/kg ketamine (Ketavet 100; Intervet Farmaceutici). Acute hindlimb ischemia was induced by removing the femoral artery, as previously described^52^. Calf perfusion measurement were carried out by VEVO 2100 Ultrasound (FUJIFILM Visualsonics Inc., Toronto, ON, Canada) in power Doppler mode under general anesthesia by 1.5–2% isoflurane (ForaneH, Abbott, Chicago, Illinois, USA). Residual calf perfusion was expressed as vascularity ratio (left ischemic/right non ischemic)^21^. Sample size was estimated by G*Power software 3.1.

### Inhibition of miR-210 in vivo

In vivo down modulation of miR-210 was carried out by intraperitoneal injection of LNA oligonucleotides against miR-210 (ANTI-210) or a scrambled control sequence (SCR) (In vivo LNAmicroRNA Inhibitors; Exiqon, Vedbæk, Denmark). The following 15mers LNA-enhanced sequences with complete phosphothioate backbone were used: ANTI-210, GCTGTCACACGCACA; SCR, CGTCTAGCCACCTAG. Wt mice underwent hind limb ischemia (day 0) and then they were randomized in two groups. After 5 days of ischemia, one group (ANTI-210) received one intraperitoneal injection of 12 mg/kg LNA-anti-miR-210 diluted in 200 µl of saline. The second group (SCR) received the same dose of scrambled sequence (SCR), as control. Both groups were sacrificed by overdose of anesthetic, 7 days after surgery. The effectiveness of ANTI-210 treatment was assessed by qPCR in each mouse on quadriceps femoris muscles or liver samples.

### Ultrasound measurement of calf perfusion

Calf perfusion measurement were performed by an experienced technician in blind, as previously described^21^. Mice were maintained under general anesthesia obtained by 1.5–2% isoflurane (ForaneH, Chicago, Illinois, Abbott) vaporized in 100% oxygen (flow: 1 l/min), in supine position. During the examination, body temperature was monitored and maintained stable at 37 °C. Measurement of perfusion were carried out by Ultrasound device VEVO 2100 (FUJIFILM Visualsonics Inc., Toronto, ON, Canada) in ischemic and non-ischemic controlateral calves, using a 2100 transducer in power Doppler mode (transmit Power 100%; center frequency 32 MHz; gate 2; pulse repetition frequency; beam angle 0; Doppler gain 35 dB; dynamic range 35 dB), 7 or 14 days after ischemia (*n* = 16–18). Residual calf perfusion was expressed as vascularity ratio (left ischemic/right non ischemic) as previously described^21^.

### Bone marrow transplantation experiments

Bone marrow transplantation was carried out as described previously^53^. 8–12 weeks old recipient Tg-210 mice (CD45.2 positive) or Wt C57BL/6N mice (CD45.1 positive, Charles River Laboratories) mice were lethally irradiated. Specifically, mice were subjected to 9.5 Gy of lethal whole-body irradiation by 2 repeated doses of X ray (450 rad each) 2 h apart, to minimize gastrointestinal toxicity. The day after, bone marrow cells from donor mice (CD45.1 or CD45.2 positive) were collected, 10 × 10^6^ cells were resuspended in 0.2 ml of PBS and injected into the tail vein of recipient mice. In particular, CD45.2-positive BM cells from Tg-210 donor mice were transplanted in lethally irradiated, CD45.1-positive Wt recipient mice (BM-Tg210/R-wt group) (Fig S5A); alternatively, BM cells from CD45.1-positive Wt donor mice were transplanted in CD45.2-positive Tg210 recipient mice (BM-wt/R-Tg210 group) (Fig. S5B). Two control groups were generated: BM-wt/R-wt mice (transplanting BM cells from CD45.2-positive Wt donor mice in CD45.1-positive Wt recipient mice) and BM-Tg210/R-Tg210 mice (transplanting CD45.2-positive BM cells from Tg-210 donor mice to CD45.2-positive Tg-210 recipient mice) (Fig S5C). Repopulation of the bone marrow was allowed for 10 weeks.

Flow cytometry was carried out on mononuclear hematocytes in order to assess the allelic variants of the pan-hematopoietic marker CD45, in the peripheral blood before BMT and in both blood and spleen samples, after BMT.

Chimeric mice (*N* = 16–18) were maintained in a dedicated pathogen-free environment and received normal chow and drinking water supplemented with Baytril antibiotic 6 ml/l (50 mg/ml Bayer, Leverkusen, Germany) for the first 2 weeks after bone marrow transplantation. Repopulation of the bone marrow was allowed for 10 weeks. To determine the engraftment of the donor bone marrow, 250–300 µl of peripheral blood were collected from the orbital sinus after anesthesia (by intraperitoneal injection of 10 mg/kg xylazine (Intervet Farmaceutici) and 100 mg/kg ketamine (Ketavet 100; Intervet Farmaceutici). 25 µl of each blood sample were analyzed by Idexx ProCyte Dx hematology analyzer (Idexx Laboratories, Westbrook, Maine, USA). The remaining blood was processed for white cells isolation and flow cytometry. Flow cytometry was carried out in order to assess the allelic variant of the pan-hematopoietic marker CD45. The following antibodies were used: anti CD45.1 (Ly5.1) (#561872, BD Pharmigen, Franklin Lakes, New Jersey, USA) and anti CD45.2 (Ly5.2) (#552950 BD Pharmigen). About 1 × 10^5^ events were acquired for each sample using a Navios flow cytometer (Beckman Coulter, Fullerton, CA), and data were analyzed using the Kaluza software (Beckman Coulter, Brea, CA). Only mice showing more than 98% engraftment and red and white cells in normal ranges were used for hindlimb ischemia experiments. Before the sacrifice, 14 days after ischemia, mice underwent a second blood collection and after the sacrifice, spleen and liver samples were harvested. Both blood and spleen samples were analyzed for CD45 allelic variants by flow cytometry, as described above. In addition, spleen and liver samples were used for qPCR analysis in order to assess miR-210 induction.

### Sample preparations

For RNA extraction, muscles were snap frozen in liquid nitrogen. For histological analysis, mice were perfused via left ventricle with PBS pH 7.5, followed by 10% buffered formalin, at 100 mm/Hg for 10 min^52^. Next, gastrocnemius muscles were harvested, fixed, paraffin embedded and sections were prepared as previously described^52^.

### Histology and morphometric analysis

Formalin-fixed paraffin-embedded consecutive sections (3 µm thickness) were dewaxed, hydrated through graded decrease alcohol series and stained for histological analysis. Hematoxylin/Eosin staining was performed using a standard protocol (Mayer’s Hematoxylin, #05-06002/L; Eosin #05-10002/L, Bio Optica, Milan, Italy). For immunofluorescence (IF) and immunohistochemistry (IHC) staining, the following antibodies were used: rat anti mouse CD45 (#14-0452-82, eBioscience, Invitrogen Carlsbad, California, USA), rat anti-mouse F4/80 (#MCA497B AbD-Serotec®, Kidlington, UK), rat anti-mouse CD68 (#MCA1957GA, Bio-Rad Kidlington, UK), goat anti-mouse MMR/CD206 (#AF2535 R&D Systems, Minneapolis, Minnesota, USA), rabbit monoclonal anti-alpha-smooth muscle actin (α-SMA) (#ab124964, Abcam, Cambridge, UK), mouse monoclonal anti-alpha-smooth muscle actin (#A2547 Sigma-Aldrich, Merck Darmstadt, Germany), rabbit polyclonal anti-Collagen V (#Ab7046, Abcam), rabbit polyclonal anti-Collagen I (#34710, Abcam), rabbit polyclonal anti-VEGF (#PA5-16754 Thermo Fisher Scientific, Waltham, Massachusetts, USA), rabbit monoclonal anti-Vimentin (#ab92547, Abcam), rabbit monoclonal anti-Smad3 phospho S423 + S425 (#Ab52903, Abcam).

Unless otherwise specified, representative images were taken by a Zeiss AxioLab-A1 microscopy (Zeiss, Oberkochen, Germany) equipped with a True Chrome HD II S camera (Tiesse Lab, Cassano d’Adda (MI), Italy) and with image analyzer ISCapture software.

For the quantification of CD45 or CD68 positive cells, 20–30 random fields/section were evaluated at ×400 magnification.

For the quantification of macrophages, images of the entire sections were obtained using an inverted point scanning confocal Leica SMD SP8 (Leica Microsystems, Wetzlar, Germany) with a 10 × /0.5 objective, by tile-scanning and automatic stitching. Images were analyzed by Cellomics ArrayScan XTI Studio Scan (Thermo Fischer Scientific) software.

Capillary density was measured counting the number of capillary profiles in hematoxylin/eosine stained sections, 30–40 random fields/section were evaluated in bright-field microscopy AxioLab-A1, (Zeiss) at ×1000 magnification^12,52^.

Arterioles were labeled by IHC using an alpha-smooth muscle actin (α-SMA) antibody (#124964 Abcam). Arterioles, were visualized at ×400 magnification and arteriolar length density (ALD) was determined as previously described^52^.

All quantifications were performed by 2 experienced histologists in blind.

Collagen fibers were stained by Sirius Red staining (Direct Red 80, #365548 Sigma) using a standard protocol. Slides were acquired with Aperio AT2 digital scanner at magnification of 200× (Leica Biosystems, Wetzlar, Germany) and analyzed by Imagescope using Color Deconvolution v9 Analysis Macro (Leica Biosystems).

Images of CD45/α-SMA, CD45/CollagenV and F4/80/CD206/α-SMA staining were taken by an inverted point scanning confocal Leica TCS SP5 (Leica Microsystems) with a HCX PL APO 40X (NA 1.25) objective and a z-stack step of 0.35 µm. The different channels were acquired in a sequential mode. Representative images are presented as Maxprojection.

### In situ hybridization

The identification of Tg-210 BM cells in chimeric mice BM-Tg210/R-wt and the identification of cells belonging to the recipient mice in BM-wt/R-Tg210 chimeric mice, was performed by in situ hybridization.

Briefly, after formalin-fixed paraffin-embedded samples preparation and pretreatment, sections underwent in situ hybridization using the RNAscope 2.5 HD Detection reagent-RED (ACD Bioscience, Newark, California, USA) or the RNAscope Multiplex Fluorescent reagent kit v2 (ACD Bioscience), using the Opal dye 650 (Akoya Bioscience, Marlborough, MA, USA) as fluorophore. A custom probe able to recognize the improved Tetracycline Repressor (iTetR) mRNA (ACD Bioscience #854171) was designed. Indeed, iTetR gene is located in the expression cassette of Tg-210 mice, but it is absent in Wt mice.

The assay was carried out following the manufacturing’s instruction, performing target retrieval at 99 °C for 20’ in histological bath and Proteinase plus treatment at 40 °C for 30’. Fast red stained representative images were taken in bright-field microscopy (Zeiss AxioLab-A1) at 400× magnification.

For the identification of Tg-210 BM derived cells expressing α-SMA in chimeric mice, fluorescent RNAscope assay for iTetR was followed by Immunofluorescence staining with anti-α-SMA antibody (Abcam #ab124964). Images of iTetR/α-SMA positive cells were taken with a 40× (NA 1.35) objective on a DeltaVision deconvolution microscope (Applied Precision, Bratislava, Slovakia) with a complete Z-series in µm sections (0.32 µm step size), deconvolved and projected (Max Projection).

### Murine bone marrow-derived macrophages isolation and culture

Bone marrow-derived macrophages were obtained and cultured as previously described^54^. Briefly, femur and tibia of Wt or Tg-210 mice were flushed with sterile RPMI medium + 10% FBS. Collected cells were filtered with a 40 µm cell strainer, centrifuged, and washed. 10 × 10^6^ cells were cultured in 10 mm-Petri dish (non-tissue culture treated, bacterial grade) in 6 ml of Differentiation Medium containing DMEM high glucose (Sigma #D6546) supplemented with 20% FBS (Euroclone #ECS1102D), 1 µg/ml doxycycline hydrochloride (Sigma #D3447) and 30% L929-conditioned medium (9.4 × 10^5^ L929 cells grown for 7 days in DMEM, 10% FBS, 1% HEPES), as source of macrophage-colony stimulating factor M-CSF (Differentiation medium)^54^. At day 3, three ml of additional Differentiation Medium were added to the plates and cultured until day 7 without changing medium. At day 7 cells were harvested by Trypsin/EDTA (Euroclone #ECB3052D) and 1 × 10^6^ cells were cultured in cell culture-treated 60 mm-plates in Differentiation medium for further 7 days (time 7 + 7) for RNA isolation. In addition, 1 × 10^5^ cells were cultured in 8 well-glass chamber slides in Differentiation Medium for 1 or 7 days for Immunofluorescence staining (time 7 + 1 and 7 + 7) (Supplementary Fig. 11). In the experiments in which the TGF-β type-1 receptor was inhibited, cells were grown for 7 days in the presence of DMSO or in the presence of 1 µM TGF-β type-1 receptor inhibitors SB431542 or A-83-01 (Tocris, Bristol UK, #1614 and #2939, respectively).

The purity of the culture was assessed at day 7 + 1 by IF staining for CD68, CD206, α-SMA and Collagen I, using the following antibodies: rat anti-mouse CD68 (Bio-Rad MCA1957GA), mouse anti-alpha smooth muscle actin (Sigma #A2547), rabbit monoclonal anti-alpha-smooth muscle actin (α-SMA) (Abcam #ab124964), rabbit polyclonal anti-Collagen I (Abcam #ab34710). The same antibodies were used for IF staining at day 7 + 7.

Chamber slides were examined using a Zeiss AxioLab-A1 fluorescence microscope with image analyzer ISCapture software. For quantification experiments, 25 random images of each chamber were acquired under ×400 magnification.

### miRNA and mRNA quantification

Total RNA was extracted from cells and tissue using TRIzol (ThermoFisher Scientific) and the TissueLyser system (Qiagen, Hilden, Germany). miRNA and mRNA levels were analyzed using the TaqMan qPCR assay (ThermoFisher Scientific) and the SYBR-GREEN qPCR method (Promega, Madison, Wisconsin, USA), respectively, and quantified with the Step-One plus real-time PCR System (ThermoFisher Scientific), as previously described^21^. Primers used for qPCR are listed in Table S1 (Supplementary materials). miRNA and mRNA relative expression was calculated using the comparative Ct method (2−Delta Delta Ct)^55^ and the expression values were normalized to miR-16 and RPL13 levels, respectively, both not modulated by ischemia or miR-210^12,21^.

## Supplementary information

Supplementary Materials and Methods

Supplementary figure S1

Supplementary figure S2

Supplementary figure S3

Supplementary figure S4

Supplementary figure S5

Supplementary figure S6

Supplementary figure S7

Supplementary figure S8

Supplementary figure S9

Supplementary figure S10

Supplementary figure S11

Supplementary figure S12

Supplementary figure S13

Supplementary figure S14

Supplementary figure S15

## Data Availability

All data generated or analyzed during this study are included in this published article and its Supplementary information files.
