# Correction: Hypoxia-induced miR-210 modulates the inflammatory response and fibrosis upon acute ischemia

**DOI:** 10.1038/s41419-021-03808-3

**Published:** 2021-05-18

**Authors:** Germana Zaccagnini, Simona Greco, Marialucia Longo, Biagina Maimone, Christine Voellenkle, Paola Fuschi, Matteo Carrara, Pasquale Creo, Davide Maselli, Mario Tirone, Massimiliano Mazzone, Carlo Gaetano, Gaia Spinetti, Fabio Martelli

**Affiliations:** 1 Laboratory of Molecular Cardiology, IRCCS Policlinico San Donato, 20097 San Donato Milanese, Milan, Italy; 2 Laboratory of Stem Cells for Tissue Engineering, IRCCS Policlinico San Donato, 20097 San Donato Milanese, Milan, Italy; 3 King’s College London, School of Cardiovascular Medicine and Sciences, BHF Center of Research Excellence, London, UK; 4 Division of Genetics and Cell Biology, Chromatin Dynamics Unit, San Raffaele University, 20132 Milan, Italy; 5 Laboratory of Tumor Inflammation and Angiogenesis, Center for Cancer Biology (CCB), VIB, and Department of Oncology, KU Leuven, 3000 Leuven, Belgium; 6 Laboratorio di Epigenetica, Istituti Clinici Scientifici Maugeri IRCCS, via Maugeri 4, 27100 Pavia, Italy; 7 Laboratory of Cardiovascular Research, IRCCS MultiMedica, 20138 Milan, Italy

**Keywords:** Cardiovascular diseases, Inflammation

Correction to: *Cell Death and Disease*

10.1038/s41419-021-03713-9 published online 01 May 2021

The original version of this article unfortunately contained a mistake. The author names were given in the wrong order (family name) (given name) and were therefore tagged incorrectly. The authors apologize for the mistake. The original article has been corrected. The correct names are given below (given name) (family name):

Germana Zaccagnini, Simona Greco, Marialucia Longo, Biagina Maimone, Christine Voellenkle, Paola Fuschi, Matteo Carrara, Pasquale Creo, Davide Maselli, Mario Tirone, Massimiliano Mazzone, Carlo Gaetano, Gaia Spinetti, Fabio Martelli

