# Supplementary material for: Hypoxia-induced miR-210 modulates the inflammatory response and fibrosis upon acute ischemia
Source: Cell Death Dis. 2021 May 1;12(5):435. doi: 10.1038/s41419-021-03713-9 (PMC8088433; doi:10.1038/s41419-021-03713-9)
Supplement: Supplementary file 1 — Supplementary Materials and Methods [file 41419_2021_3713_MOESM1_ESM.docx]

**Hypoxia-induced miR-210 modulates the inflammatory response and fibrosis upon acute ischemia**

Zaccagnini Germana^1*^, Greco Simona^1^, Longo Marialucia^1^, Maimone Biagina^1^, Voellenkle Christine^1^, Fuschi Paola^1^, Carrara Matteo^1^, Creo Pasquale^2^, Maselli Davide^3^, Tirone Mario^4^, Mazzone Massimiliano ^5^, Gaetano Carlo ^6^, Spinetti Gaia^7^, Martelli Fabio^1#^

^1^Laboratory of Molecular Cardiology, IRCCS Policlinico San Donato, 20097 San Donato Milanese, Milan, Italy;

^2^Laboratory of stem cells for tissue engineering, IRCCS Policlinico San Donato, 20097 San Donato Milanese, Milan, Italy

^3^King's College London, School of Cardiovascular Medicine and Sciences, BHF Center of Research Excellence, London, United Kingdom

^4^Division of Genetics and Cell Biology, Chromatin Dynamics Unit, San Raffaele University, Milan, Italy.

^5^ Lab of Tumor Inflammation and Angiogenesis, Center for Cancer Biology (CCB), VIB, and Department of Oncology, KU Leuven, 3000 Leuven, Belgium.

^6^ Laboratorio di Epigenetica, Istituti Clinici Scientifici Maugeri IRCCS, via Maugeri 4, 27100 Pavia, Italy.

^7^Laboratory of Cardiovascular Research, IRCCS MultiMedica, 20138 Milan, Italy

# **Corresponding author Dr. Fabio Martelli**

Via Morandi, 30 20097 San Donato Milanese, (MI) Italy

[fabio.martelli@grupposandonato.it](mailto:fabio.martelli@grupposandonato.it)

Phone number: +390226437762 or +390252774533

**Running Title:**

**miR-210 modulates inflammation and fibrosis**

**Key words**

**miR-210, hindlimb ischemia, bone marrow transplantation, inflammation, fibrosis**

# SUPPLEMENTAL DATA

### Table S1

The primers listed below were used for qPCR analysis:

| Gene Name | Forward | Reverse |
| --- | --- | --- |
| Acta2  (NM_007392) | CGGAAGCGTTCGTTTCCAAT | GAGCGTGAGATTGTCCGTGA |
| Ccl4  (NM_013652) | TTCCTGCTGTTTCTCTTACACCT | CTGTCTGCCTCTTTTGGTCAG |
| Ccr5  (NM_009917) | TTTTCAAGGGTCAGTTCCGAC | GGAAGACCATCATGTTACCCAC |
| Col1a1  (NM_007742) | CTACTACCGGGCCGATGATG | AGTGGCACATCTTGAGGTCG |
| Col3a1  (NM_009930) | AGGCAACAGTGGTTCTCCTG | CCTTCTCTCCAGGCTGTCCA |
| Cxcl10  (NM_021274) | CCAAGTGCTGCCGTCATTTTC | GGCTCGCAGGGATGATTTCAA |
| Cxcl9  (NM_008599) | GCCGAGGCACGATCCACTAC | CTAGGCAGGTTTGATCTCCGTTC |
| Myh10  (NM_175260) | CACTGGGATGACTGAGACCG | ACGAAGTTGGGGTTGGTGTT |
| Tcirg  (NM_001136091) | GTTCCGGAGTGAAGAGGTGG | GCTGATTGCCACGAACATCC |

## Figure legends

### Figure S1

**Schematic representation of gain- and loss-of function experiments. A.** Gain of function: schematic representation of miR-210 overexpression. Wt and Tg-210 untreated mice (UT) underwent surgery at time 0. After 4 days and until the end of the study, both groups were fed with pellets of food containing doxycycline to induce miR-210 over-expression or as control. Mouse were analyzed 3 days later (at 7 days of ischemia) by power Doppler and sacrificed for sample harvesting and histological analysis. **B.** Loss of function: schematic representation of miR-210 blocking in hindlimb ischemia. WT mice underwent hindlimb ischemia (day 0) and, at day 5, were randomized into two groups for further treatments. In one of the groups, miR-210 function was blocked by systemic administration of ANTI-210; the second group received a SCR sequence and was used as control. Mouse were analyzed 3 days later (at 7 days of ischemia) by power Doppler and sacrificed for sample harvesting and histological analysis.

### Figure S2

### Heat map of validated differentially expressed genes. The level of the indicated genes was measured in SCR and ANTI-210 ischemic gastrocnemius muscles by microarrays analysis 7 days after ischemia and was validated by qPCR, (n=11/group). The heat map shows average expression levels where green and red colours indicate down- or upregulation, respectively.

### Figure S3

**Macrophages density is increased upon miR-210 blocking.** **A**. Representative immunofluorescence staining for CD68 (red), a pan-macrophage marker, in gastrocnemius muscle sections of SCR and ANTI-210 treated mice, 7 days after ischemia. Nuclei are stained by Hoechst (Blue). Images are presented as merge. Magnification 200x, calibration bar 100 µm **B.** The box plot shows the quantification of positive cells (n=4; test T *P<0.02).

### Figure S4

**Assessment of the CD45 allelic variant of Tg210 mice.** Representative images of flow cytometry analysis carried out on peripheral blood of Tg-210 mice and WT littermates to assess the allelic variant of the hematopoietic marker CD45. The graphs show that Tg210 hematopoietic cells expressed the CD45.2 allele.

### Figure S5

**Schematic representation of BMT experimental plan.** **A.** BM cells were harvested from Tg-210 untreated donor mice (CD45.2-positive) and injected in the tail vein of lethally irradiated recipient WT mice (CD45.1-positive) generating the chimeric mice BM-Tg210/R-wt. **B.** BM cells were harvested from WT mice (CD45.1 positive) and injected in the tail vein of lethally irradiated recipient Tg-210 generating the chimeric mice BM-wt/R-Tg210 **C.** Two control groups were generated: BM-wt/R-wt chimeric mice were obtained injecting CD45.2 BM cells in the tail vein of lethally irradiated recipient WT mice (CD45.1-positive); BM-Tg210/R-Tg210 chimeric mice were obtained injecting CD45.2 BM cells in the tail vein of lethally irradiated recipient Tg-210 mice (CD45.2-positive).

Ten weeks later, blood samples were harvested in order to assess complete hematopoietic repopulation and immunological reconstitution. Reconstituted mice underwent hindlimb ischemia and four days later miR-210 was induced by doxycycline administration. At day 14, calf perfusion was measured and mice were sacrificed for histological analysis. Analysis was performed at day 14, as preliminary experiments performed at day 7 indicated a slower vascular regeneration process in BM-transplanted mice compared to non-irradiated mice.

### Figure S6

### BM-Tg210/R-wt chimeric mice show fibrotic regions enriched in myofibroblasts. The presence of myofibroblasts was assessed by IF staining for α-SMA, Vimentin and Collagen V on serial sections of ischemic gastrocnemius muscles of BM-wt/R-wt, BM-Tg210-R-Tg210, BM-Tg210-R-wt and BM-wt/R-210 chimeric mice at day14. Magnification 200x, calibration bar 50 µm.

### Figure S7

### No TGF-β1-Smad3-p pathway activation in ischemic, non-chimeric, gastrocnemius muscles. Representative image of anti-phospho-Smad3 IHC on ischemic gastrocnemius muscle sections of SCR treated mice at day 7. Positive nuclei are stained in brown/black (Smad3-P+Hematoxylin), negative nuclei are stained in blue (hematoxylin alone). Magnification 400x, calibration bar 50 µm. Insets show magnifications of the indicated areas.

### Figure S8

### iTetR *in situ* hybridization allow identifying Tg-210 cells. Representative images of *in situ* hybridization for iTetR mRNA on ischemic gastrocnemius muscle sections of Wt e Tg-210 mice, 7 days after ischemia. Positive cells show nuclear and perinuclear purple dots (fast red). Nuclei are stained in blue (hematoxylin). Magnification 400X, calibration bar 50 µm.

### Figure S9

### BM-Tg210/R-wt chimeric mice show BM cells (CD45+) infiltrated in the ischemic tissue, which express α-SMA and/or Collagen V. Representative double immunofluorescence staining for CD45/α-SMA (A) and CD45/Collagen V (B) in ischemic gastrocnemius muscles sections of BM-wt/R-210 and BM-Tg210-R-wt chimeric mice at day 14. Merge included the nuclear staining Hoechst (blue). Representative images are presented as Maxprojection. Magnification 40X, calibration bar 50 µm. Insets show magnifications of the indicated areas.

### Figure S10

### Characterization of BM cells in BM-wt/R-Tg210 ischemic muscles.

Ischemic gastrocnemius muscles sections of BM-wt/R-Tg210 mice were stained with F4/80 (green), CD206 (red) and α-SMA (white). (A) Representative images of the triple immunofluorescence staining. The white staining indicates the tunica media of an artery. In keeping with the low inflammatory levels in these mice, no macrophages were found. Only few chimeric BM-wt/R-Tg210 mice show some residual inflamed regions in their ischemic muscles, represented in (B). The white signals indicate the tunica media of arteries, the green signals indicate F4/80 positive macrophages (bottom inset). Double-positive F4/80/CD206 macrophages are shown in the top inset. No triple positive cells were found. All merge panels include Hoechst (blue) nuclear staining. Representative images are presented as Maxprojections. Magnification 63x0.5, calibration bar 50µm. Insets show magnifications of the indicated areas.

### Figure S11

**Schematic representation of murine BM-derived macrophages isolation and culture**. Femur and tibia of WT or Tg-210 mice were flushed with sterile RPMI 1640 medium + 10% FBS. Collected cells were cultured in Petri dishes (non-tissue culture treated, bacterial grade) in DMEM supplemented with 20% FBS and 30% L929-conditioned medium as source of M-CSF (Differentiation medium). At day 7 BMDM were harvested and cultured for additional 7 days (T 7+7) in cell culture dishes for RNA extraction and qPCR analysis or in chamber slides of glass for 1 or 7 additional days (T 7+1 or 7+7) for Immunofluorescence staining. This image was in part realized by BioRender.

### Figure S12

### Characterization of BM-derived cells at day 7+1. BM derived cells express macrophages markers and are negative for α-SMA and Collagen I at day 7+1. A. Representative immunofluorescence staining of the pan-macrophages marker CD68 on BM derived Wt and Tg-210 cells after 7+1 days of culture in differentiation medium. Magnification 400x, calibration bar 50 µm. B. Dots plots represent the percentage of CD68 positive macrophages/total nuclei (N=4 different experiments). C. Representative immunofluorescence staining of the M2 macrophages marker CD206 on BM-derived Wt and Tg-210 cells after 7+1 days of culture in differentiation medium. Magnification 400x, calibration bar 50 µm. D. Representative double immunofluorescence staining of α-SMA and Collagen I on BM derived Wt and Tg-210 cells after 7+1 days of culture in differentiation medium. Magnification 400x, calibration bar 50 µm.

### Figure S13

### Tg-210 BM-derived macrophages express significantly higher levels of fibrosis markers compared to Wt. The mRNA level of the fibrosis markers ACTA2, Coll1A1, Coll3A1 and MYH10 was measured by qPCR. Dots plots represent the mRNA level in Tg-210 cells expressed as Log_2_ fold change versus Wt. N=9, ***P<0.0003; **P=<0.002.

### Figure S14

### TGF-β1 pathway inhibition completely abrogated the pro-fibrotic ability of Tg-210 macrophages. Wt and Tg-210 myofibroblasts were identified by immunofluorescence staining for α-SMA at day 7+7, after 7 days of TGFβ type-1-receptor inhibition. Two different inhibitors were used: inhibitor-A and inhibitor-SB. Dot plot represents quantification of Tg-210 myofibroblasts/total nuclei, expressed as fold change versus Wt.

### Figure S15

### Schematic representation of conclusions. Infiltrating macrophages, over-expressing miR-210 promote angiogenesis and tissue repair when the damaged recipient milieu over-express miR-210 too. On the contrary, when Tg-210 macrophages infiltrate a Wt milieu, impaired tissue repair, fibrosis and dysfunctional angiogenesis were observed. This image was in part realized by BioRender.com.
