# Supplementary figures and images for: Hypoxia-induced miR-210 modulates the inflammatory response and fibrosis upon acute ischemia
Source: Cell Death Dis. 2021 May 1;12(5):435. doi: 10.1038/s41419-021-03713-9 (PMC8088433; doi:10.1038/s41419-021-03713-9)

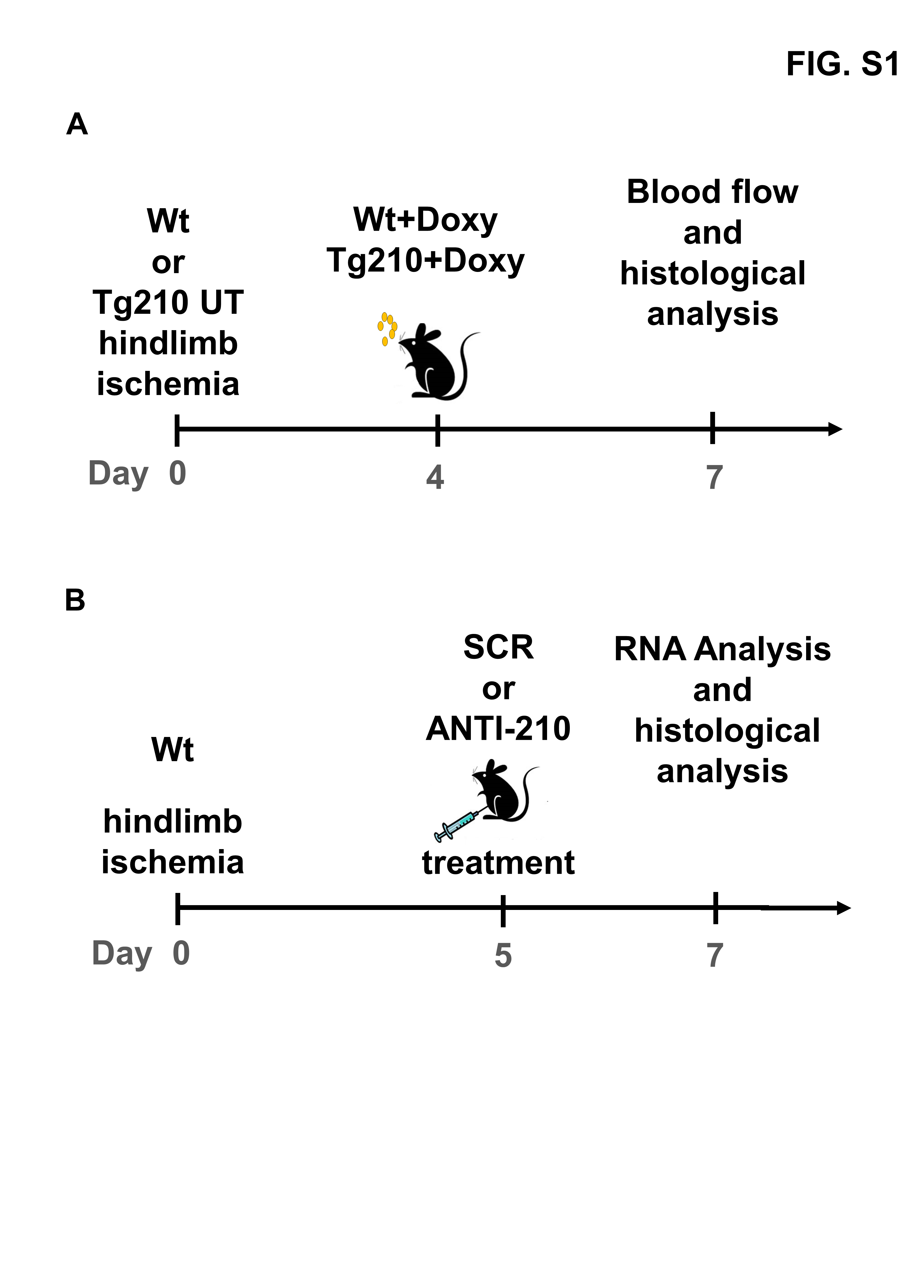

Supplement: Supplementary file 2 — Supplementary figure S1 [file 41419_2021_3713_MOESM2_ESM.tif]

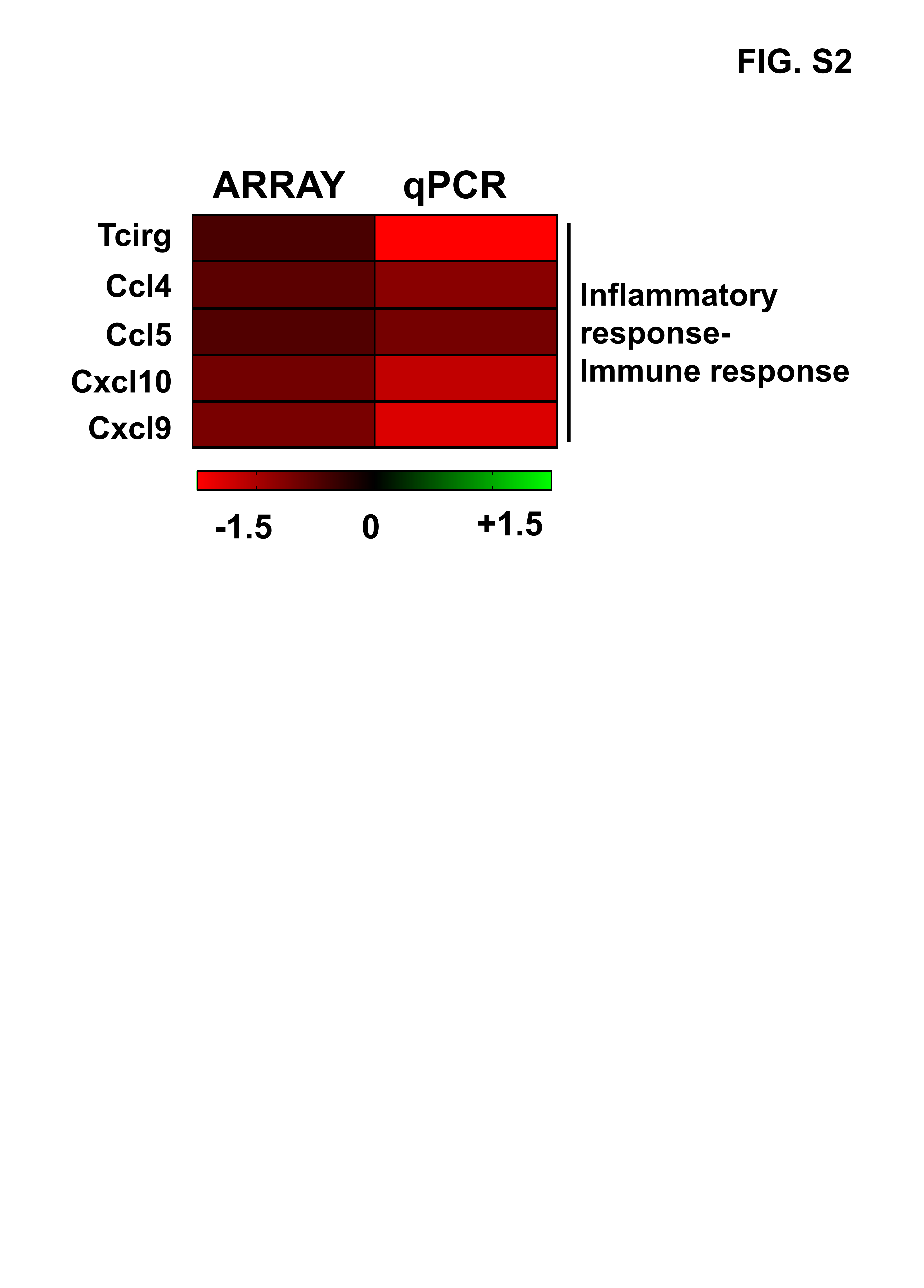

Supplement: Supplementary file 3 — Supplementary figure S2 [file 41419_2021_3713_MOESM3_ESM.tif]

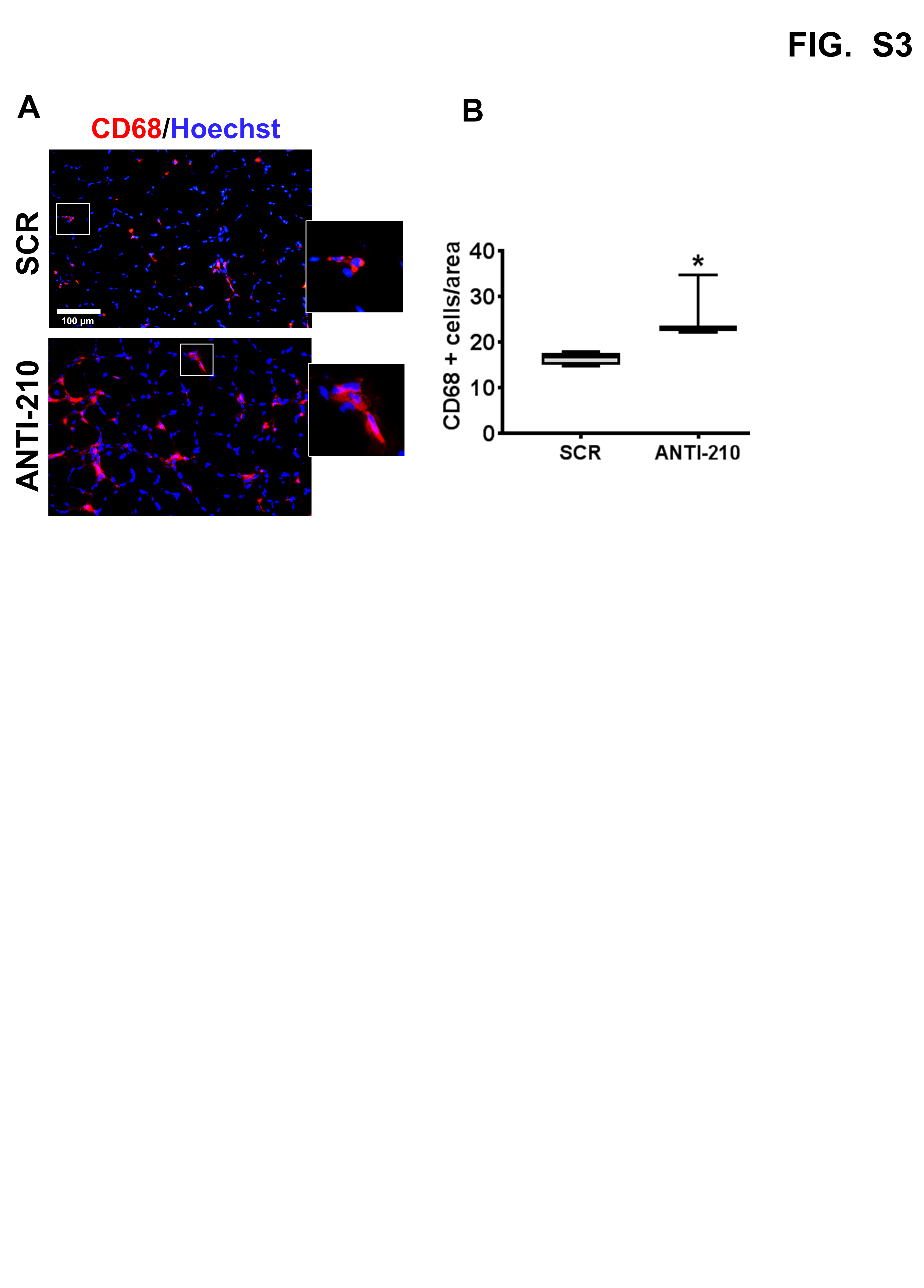

Supplement: Supplementary file 4 — Supplementary figure S3 [file 41419_2021_3713_MOESM4_ESM.tif]

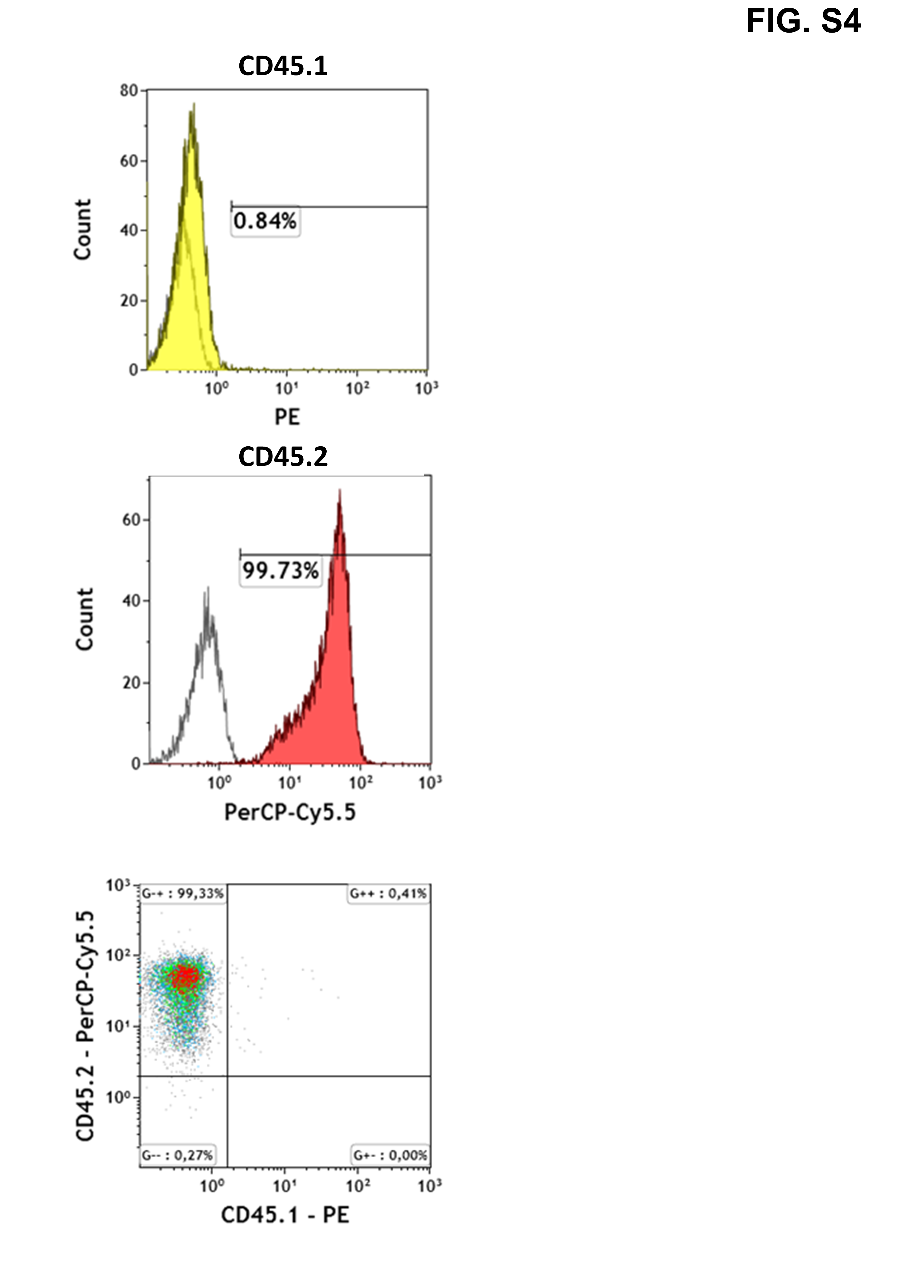

Supplement: Supplementary file 5 — Supplementary figure S4 [file 41419_2021_3713_MOESM5_ESM.tif]

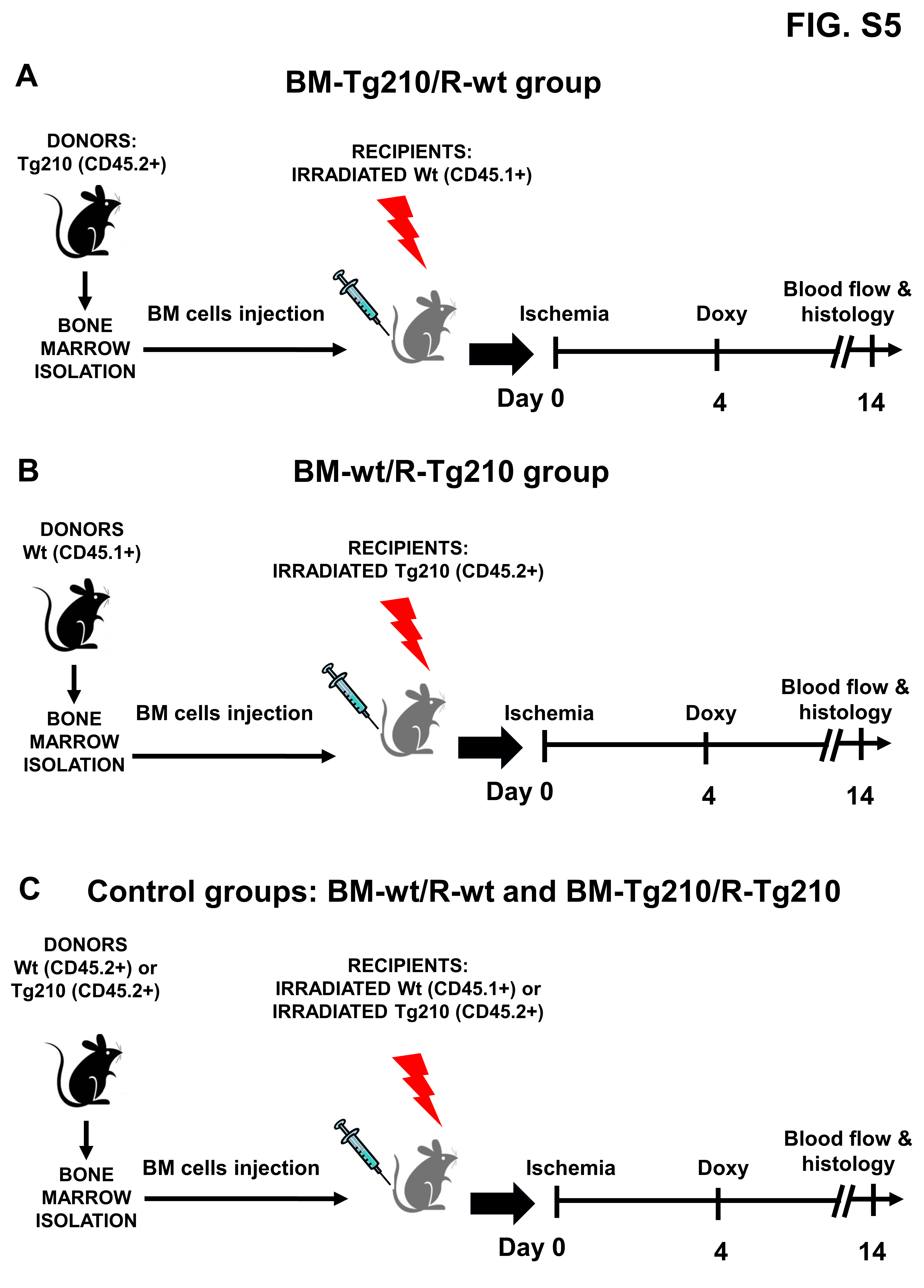

Supplement: Supplementary file 6 — Supplementary figure S5 [file 41419_2021_3713_MOESM6_ESM.tif]

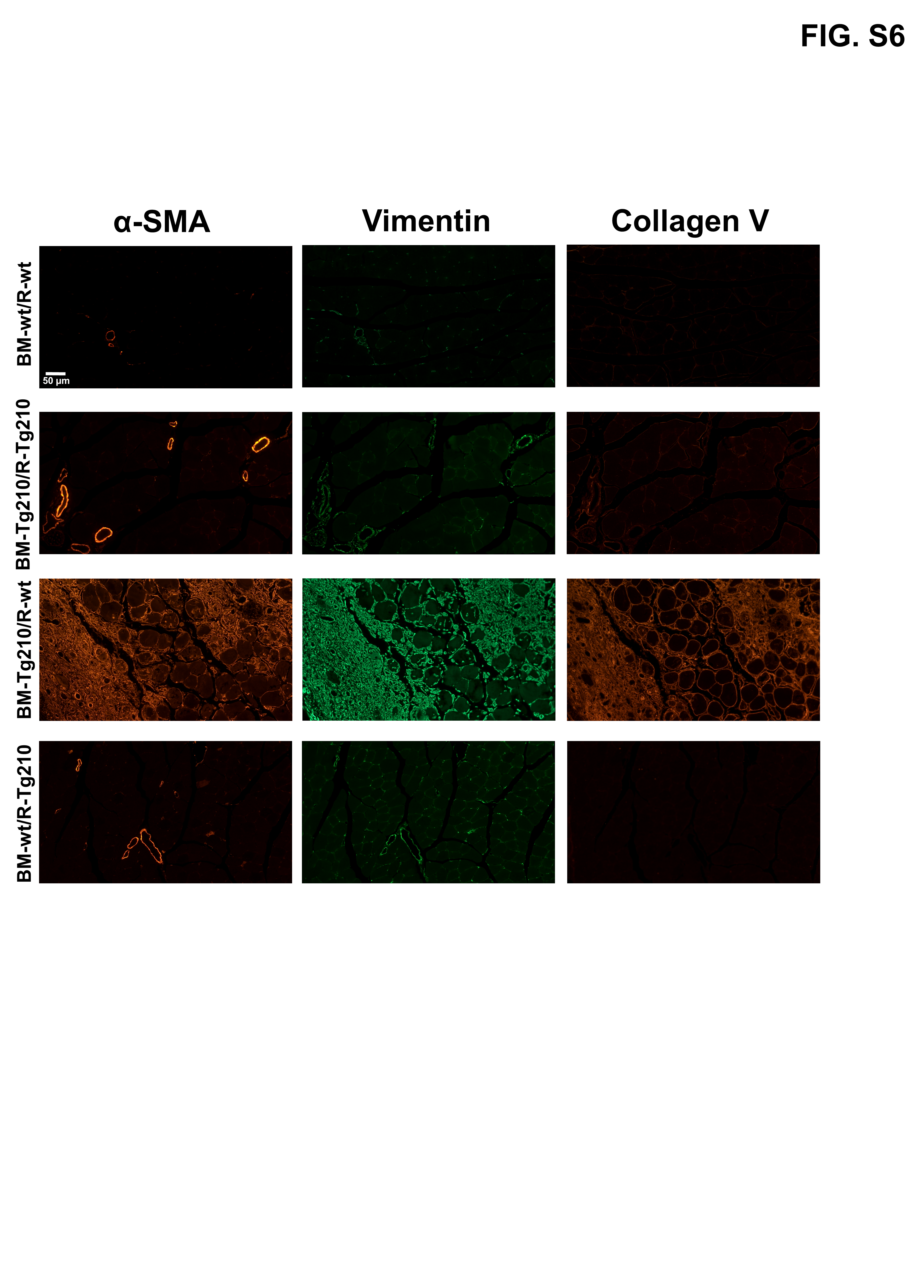

Supplement: Supplementary file 7 — Supplementary figure S6 [file 41419_2021_3713_MOESM7_ESM.tif]

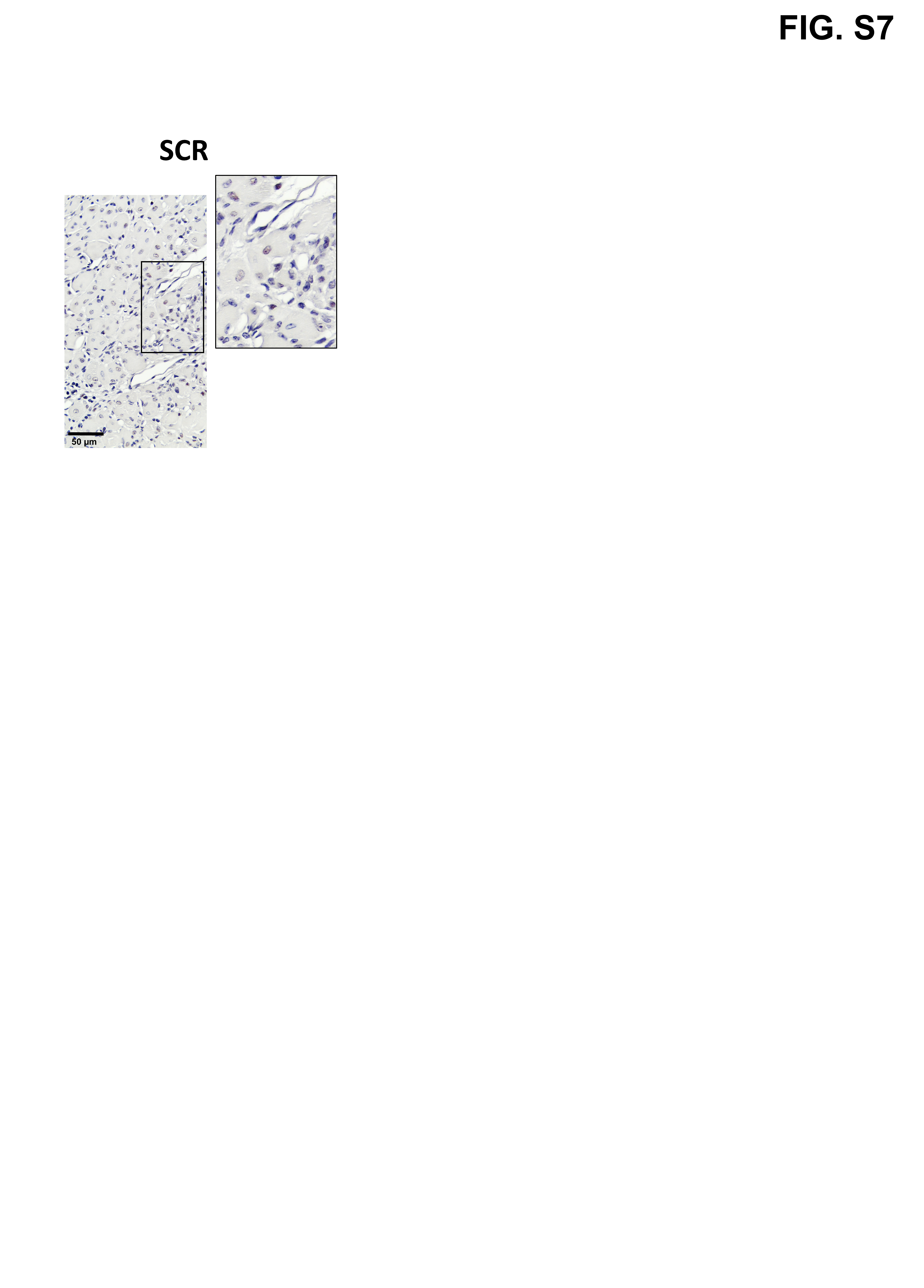

Supplement: Supplementary file 8 — Supplementary figure S7 [file 41419_2021_3713_MOESM8_ESM.tif]

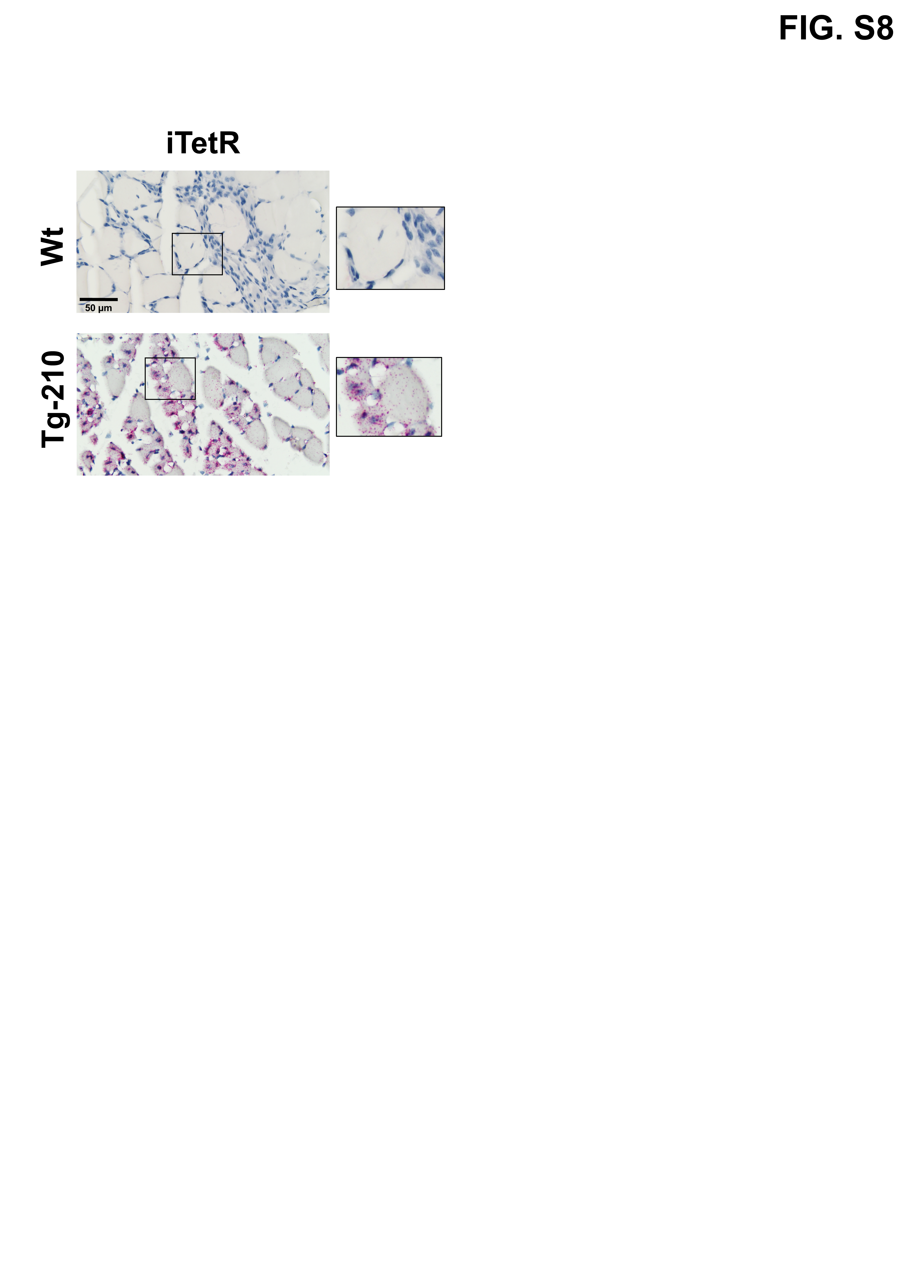

Supplement: Supplementary file 9 — Supplementary figure S8 [file 41419_2021_3713_MOESM9_ESM.tif]

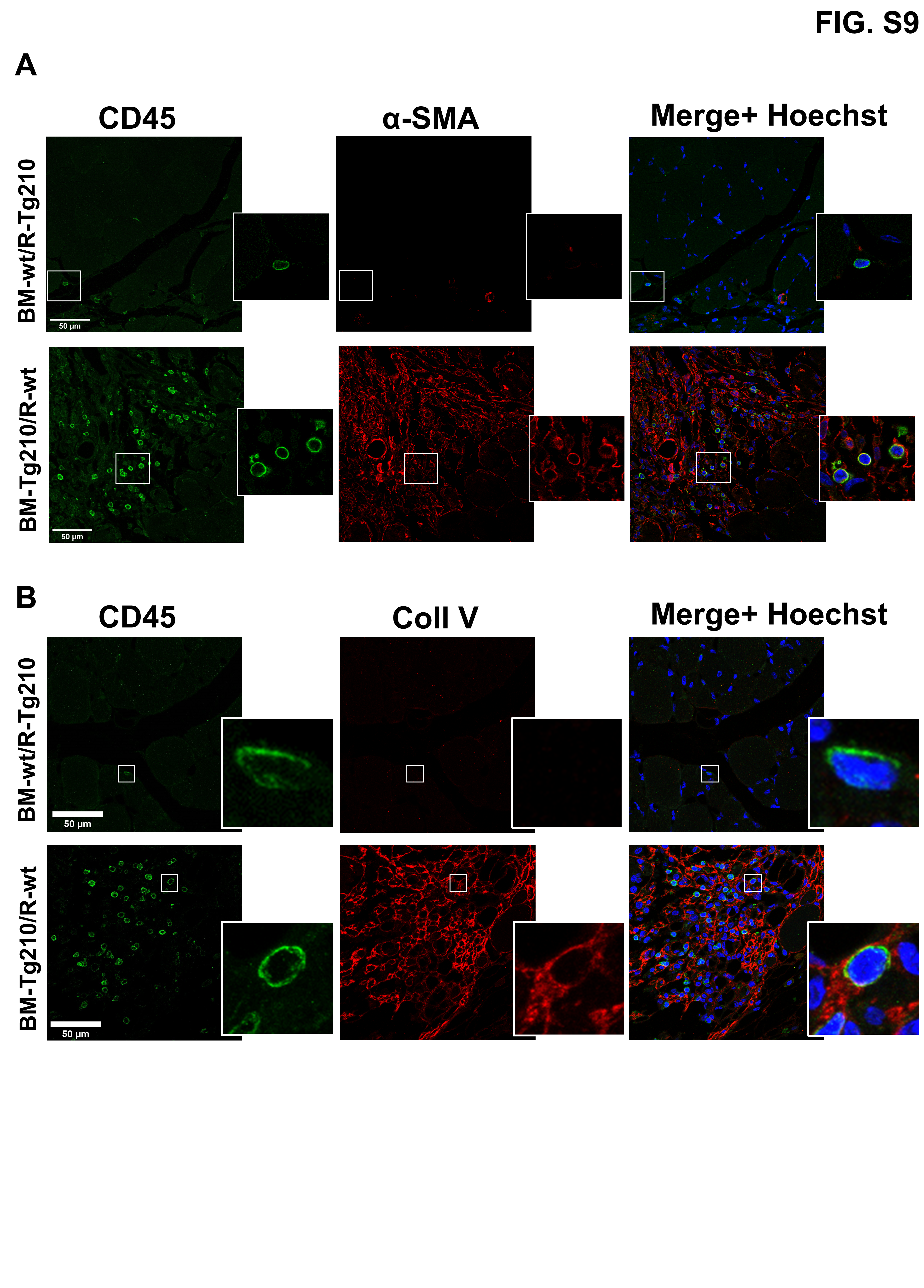

Supplement: Supplementary file 10 — Supplementary figure S9 [file 41419_2021_3713_MOESM10_ESM.tif]

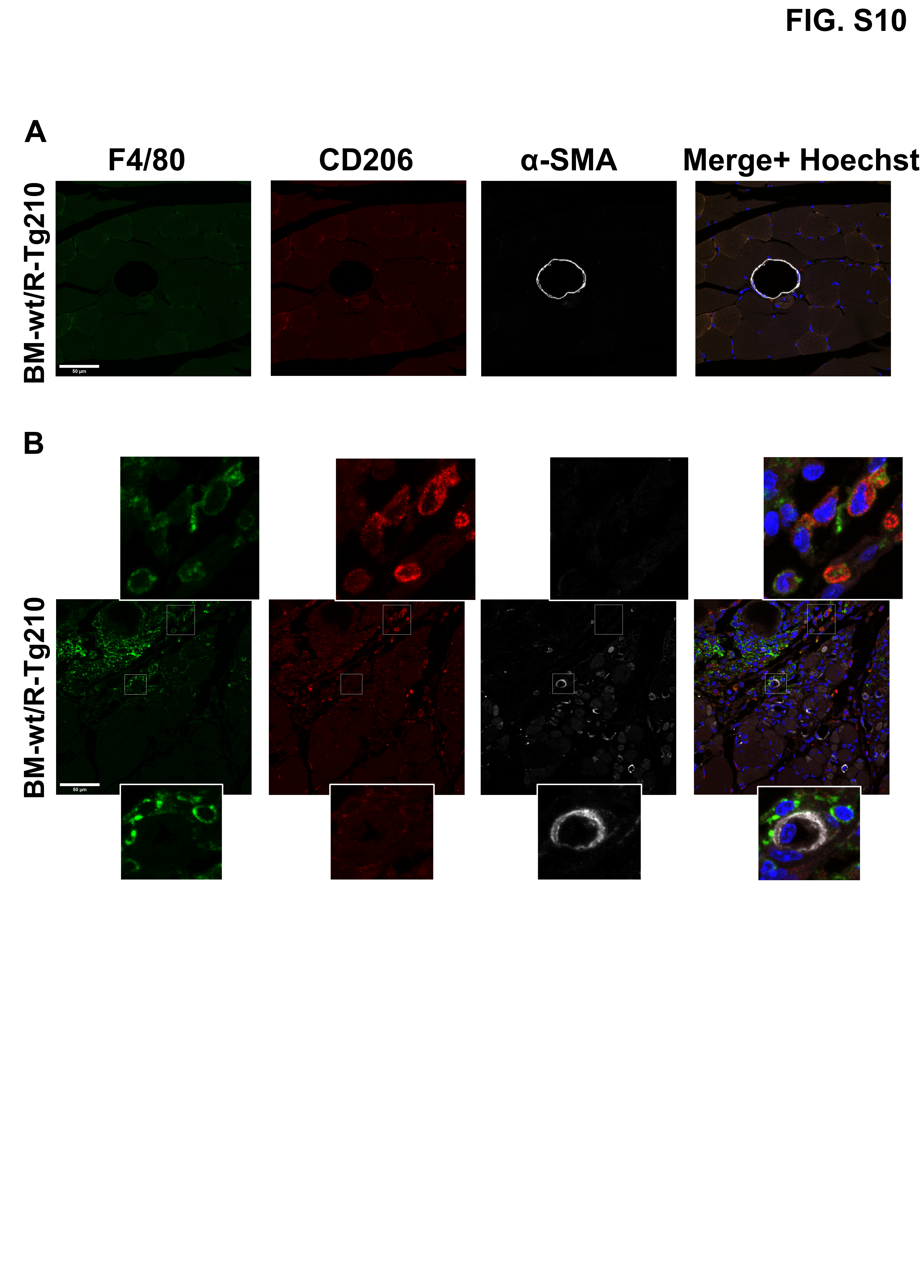

Supplement: Supplementary file 11 — Supplementary figure S10 [file 41419_2021_3713_MOESM11_ESM.tif]

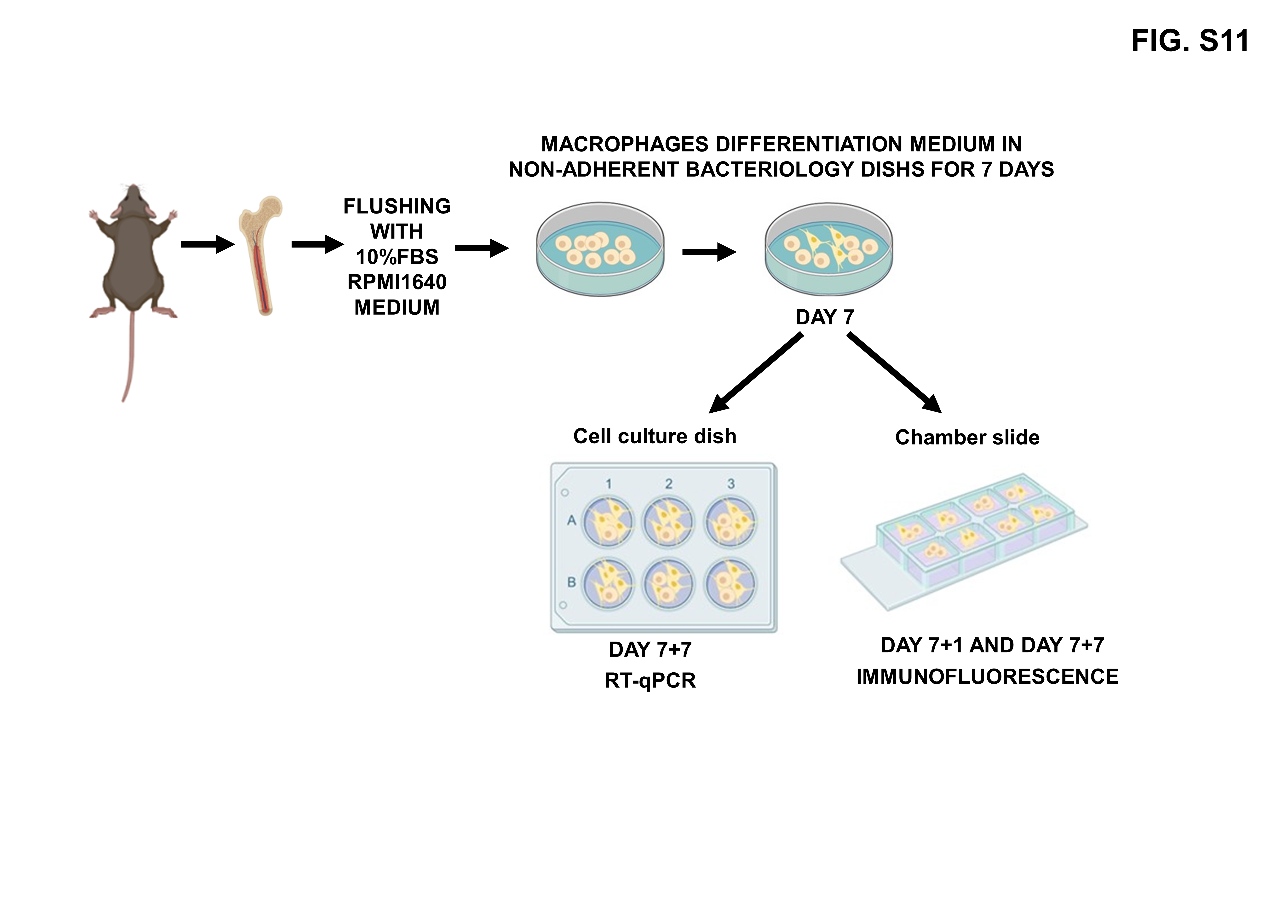

Supplement: Supplementary file 12 — Supplementary figure S11 [file 41419_2021_3713_MOESM12_ESM.tif]

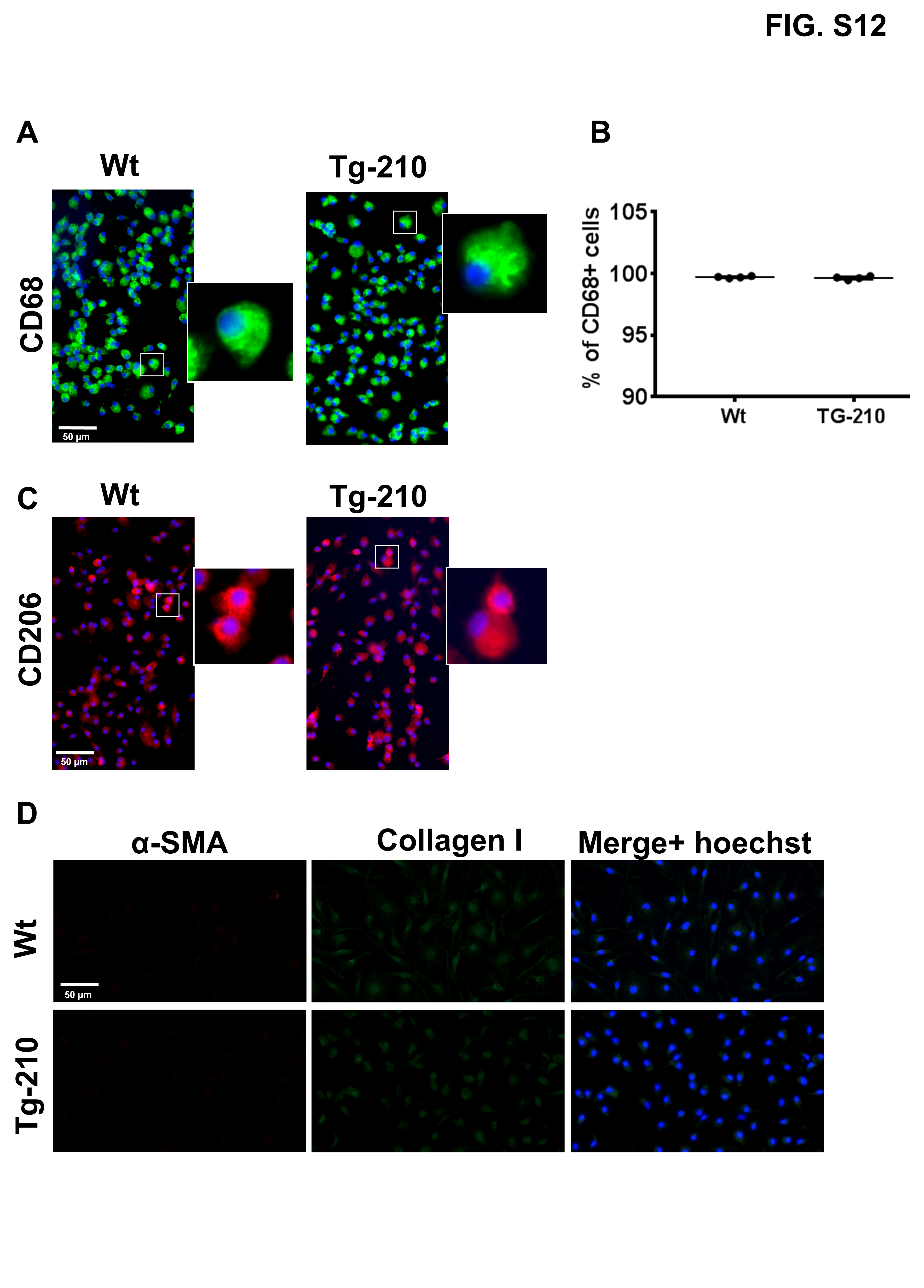

Supplement: Supplementary file 13 — Supplementary figure S12 [file 41419_2021_3713_MOESM13_ESM.tif]

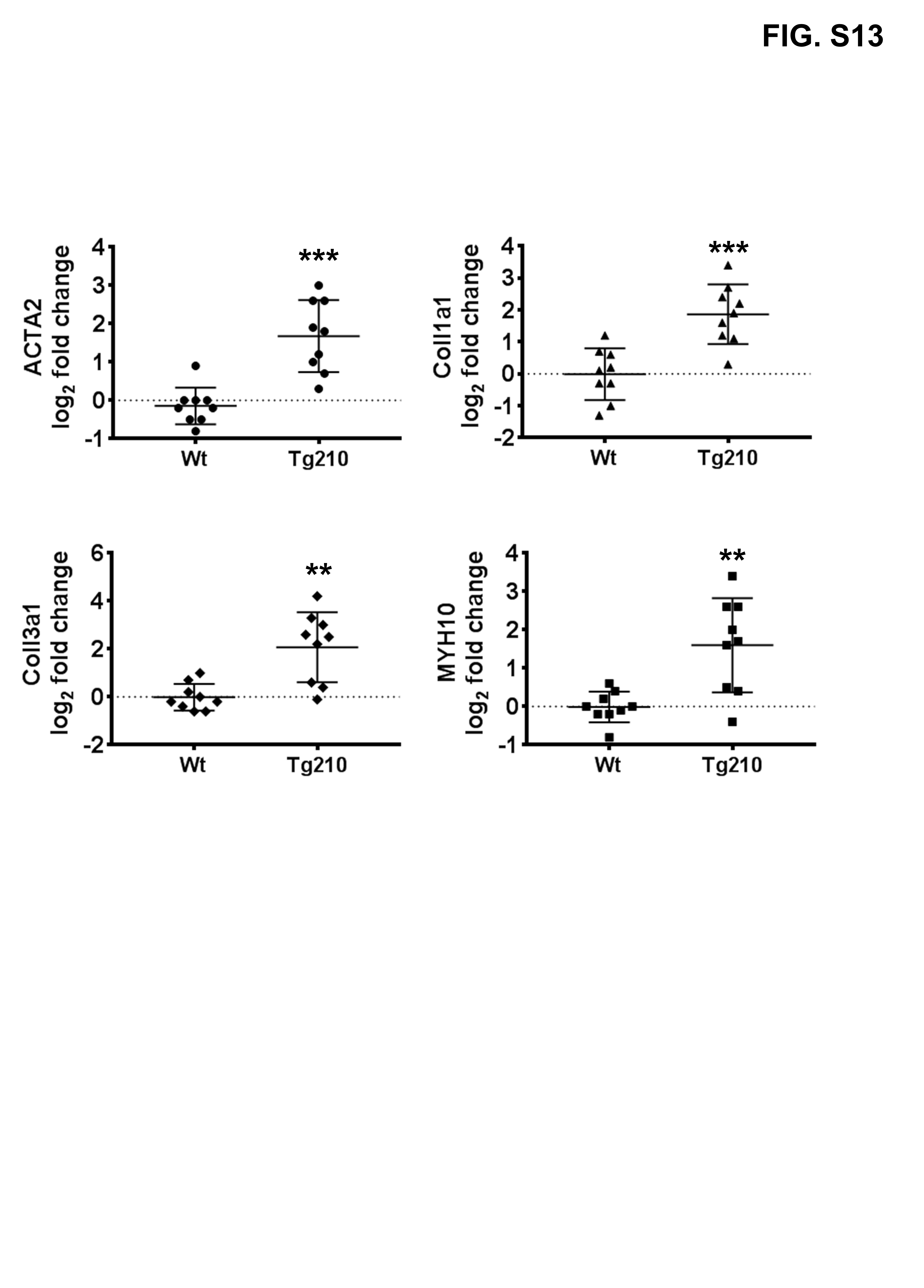

Supplement: Supplementary file 14 — Supplementary figure S13 [file 41419_2021_3713_MOESM14_ESM.tif]

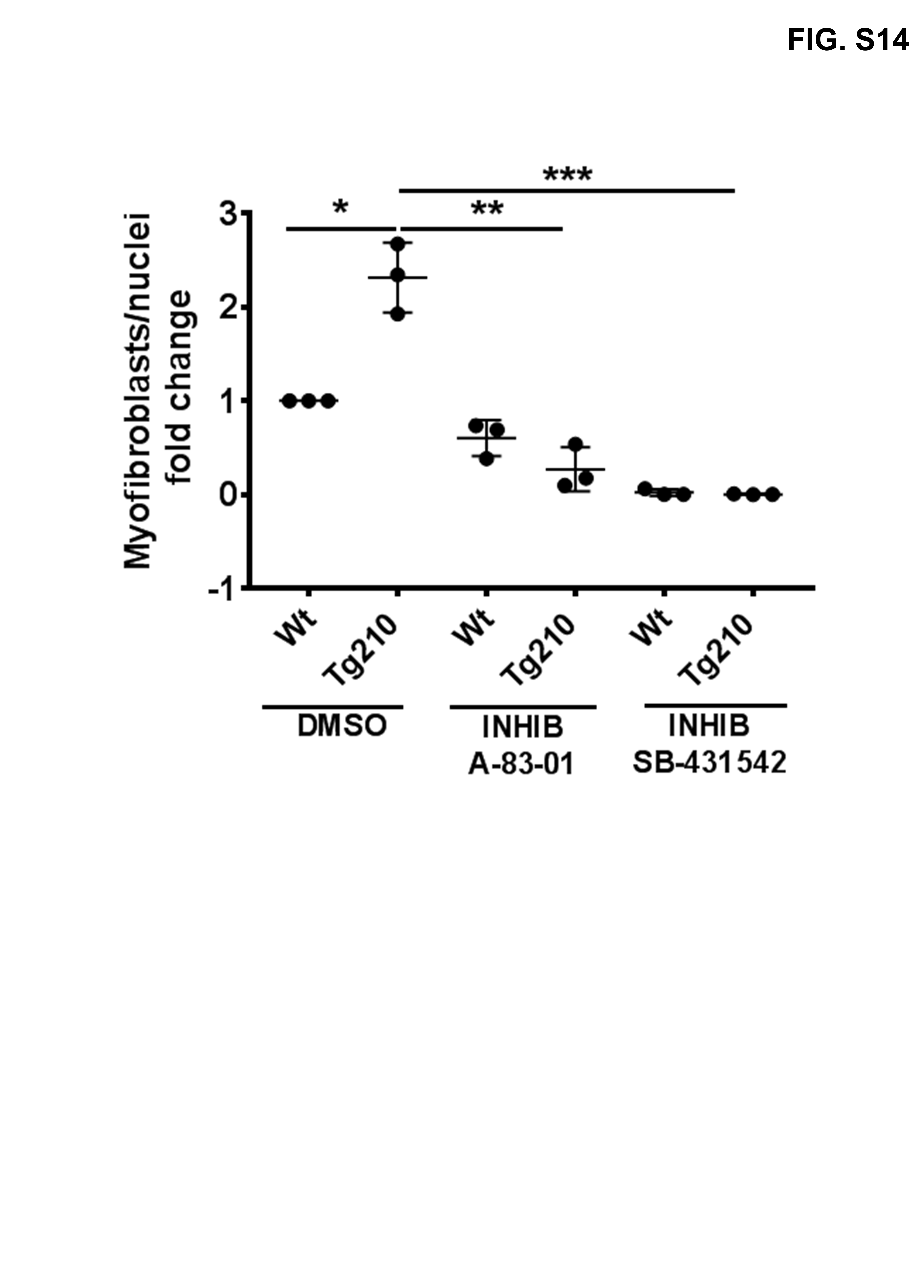

Supplement: Supplementary file 15 — Supplementary figure S14 [file 41419_2021_3713_MOESM15_ESM.tif]

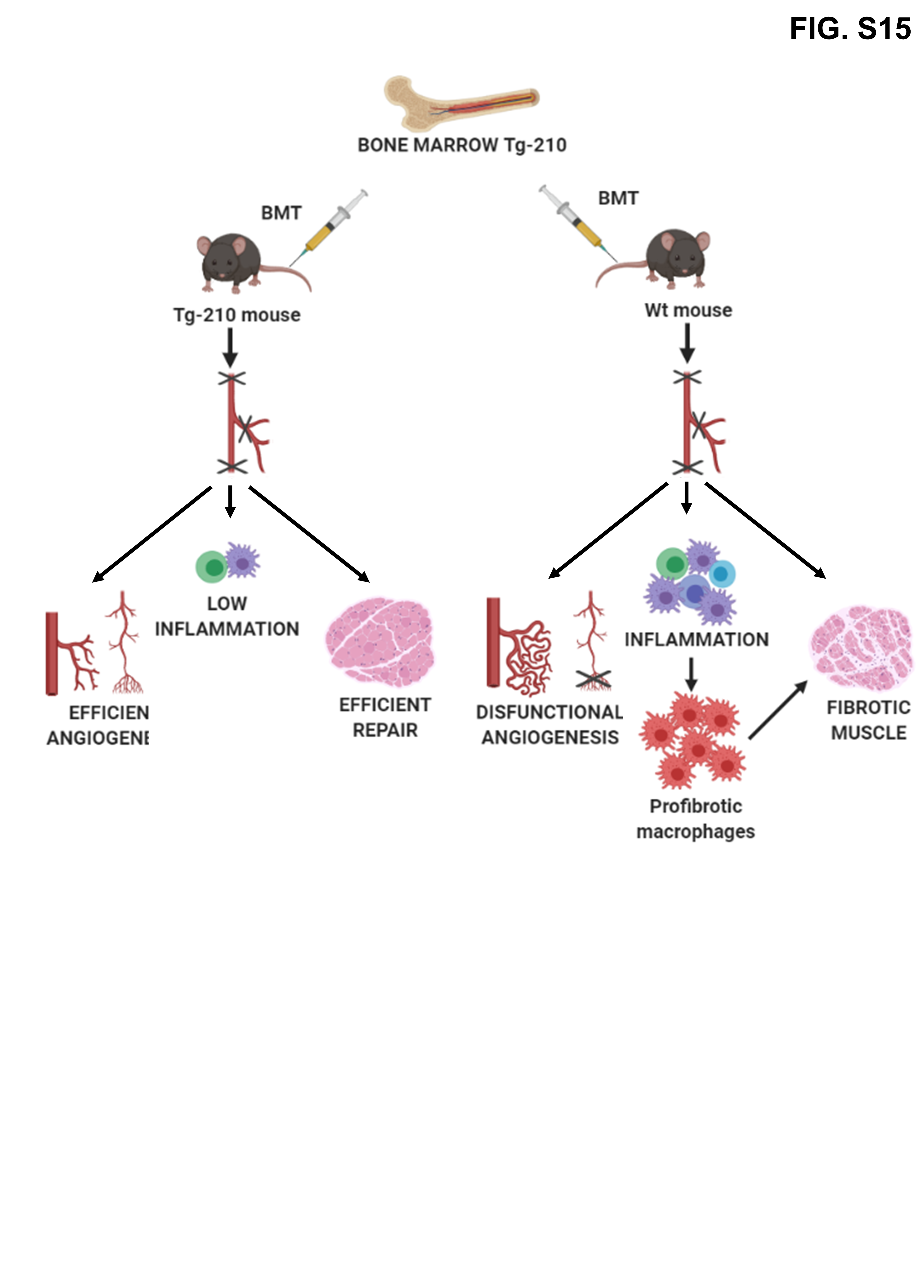

Supplement: Supplementary file 16 — Supplementary figure S15 [file 41419_2021_3713_MOESM16_ESM.tif]
